# Rationally Modified Antimicrobial Peptides from the N-Terminal Domain of Human RNase 3 Show Exceptional Serum Stability

**DOI:** 10.1021/acs.jmedchem.1c00795

**Published:** 2021-08-03

**Authors:** Daniel Sandín, Javier Valle, Belén Chaves-Arquero, Guillem Prats-Ejarque, María Nieves Larrosa, Juan José González-López, María Ángeles Jiménez, Ester Boix, David Andreu, Marc Torrent

**Affiliations:** † Department of Biochemistry and Molecular Biology, Universitat Autònoma de Barcelona, Cerdanyola del Vallès 08193, Spain; ‡ Department of Experimental and Health Sciences, Universitat Pompeu Fabra, Barcelona Biomedical Research Park, Barcelona 08003, Spain; § Departamento de Química-Física Biológica, Instituto de Química Física Rocasolano (IQFR-CSIC), Serrano 119, Madrid 28006, Spain; ∥ Servei de Microbiologia, Hospital Universitari Vall d’Hebron, Barcelona 08035, Spain; ⊥ Departament de Genètica i Microbiologia, Universitat Autònoma de Barcelona, Cerdanyola del Vallès 08193, Spain

## Abstract

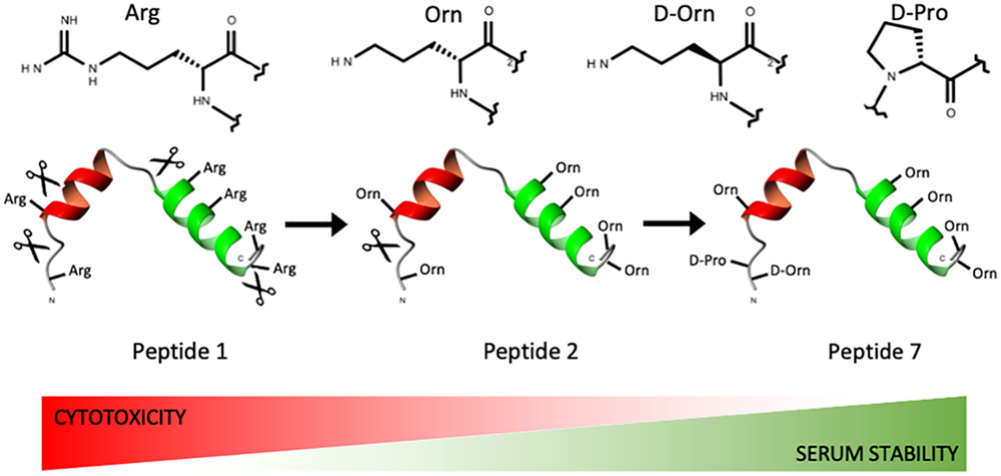

Multidrug resistance against conventional antibiotics poses an important threat to human health. In this context, antimicrobial peptides (AMPs) have been extensively studied for their antibacterial activity and promising results have been shown so far. However, AMPs tend to be rather vulnerable to protease degradation, which offsets their therapeutic appeal. Here, we demonstrate how replacing functional residues in the antimicrobial region of human RNase 3—also named eosinophil cationic protein—by non-natural amino acids increases stability in human serum. These changes were also shown to reduce the hemolytic effect of the peptides in general terms, whereas the antimicrobial activity was reasonably preserved. Digestion profiles enabled us to design new peptides with superior stability and lower toxicity that could become relevant candidates to reach clinical stages.

## Introduction

The increasing spread of antibiotic-resistant bacteria is putting our health system at risk.^1−3^ Misuse and abuse of antibiotic prescriptions in humans and the extended use in animal feeding are leading causes of antibiotic resistance.^4^ In this scenario, in addition to awareness campaigns to curb antibiotic overconsumption, new antimicrobial drugs are urgently needed.

Among the newest emergent agents against resistant bacterial infections, antimicrobial peptides (AMPs) are very promising candidates.^5,6^ AMPs are produced by many organisms to kill pathogens and modulate the host response against infections.^7−9^ Their mode of action is based on bacterial membrane destabilization in a fast and unspecific manner,^10^ although intracellular interactions (i.e., targeting protein synthesis or DNA replicating pathways) have also been reported.^11^ The human RNase A protein family includes proteins with a wide functional repertoire, besides its main ribonuclease activity.^12−53^ Antimicrobial, antihelmintic, antitumor, and cytotoxic actions have been extensively studied.^14−18^ Within this family, human RNase 3, also called eosinophil cationic protein (ECP), has a potent antimicrobial activity against both Gram-negative and Gram-positive bacteria, stronger against the former group.^19,20^ Previous studies located the antimicrobial domain of ECP on the N-terminal region,^21,22^ which could be trimmed down by structure-based design without significant loss of antimicrobial activity.^23^

In recent years, several limitations of AMPs, including industrial scale up or high production costs, have been satisfactorily addressed.^25^ Yet, two main drawbacks still preclude their transference to clinics: (i) low stability to serum proteases and (ii) cytotoxicity to host cells.^26^

**Figure 1:**
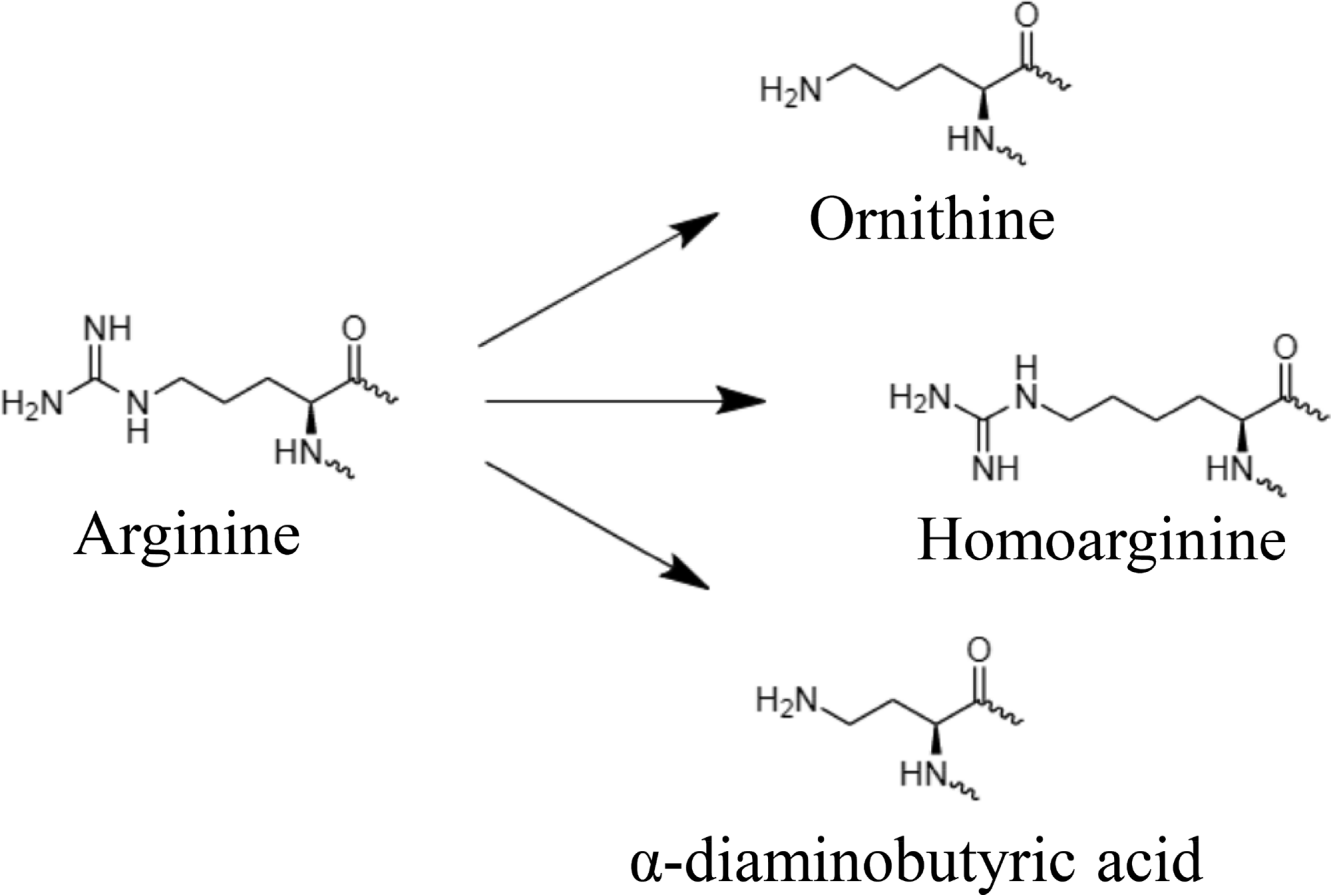
Structure of the amino acids chosen to replace Arg in the ECP-derived peptide.

Here, we designed and tested an optimized fragment from the ECP antimicrobial region (hECP30 peptide) using a rational, structure-guided design with enhanced serum stability and low cytotoxicity effects.^27,28^ First, we analyzed the digestion pattern of hECP30 and identified its most vulnerable points for protease degradation. Following Arg replacement with non-proteinogenic surrogates (Figure 1), we created a highly stable fragment with a half-life above 6 h in the presence of human serum. These peptide analogs have similar antimicrobial activity against Gram-negative bacterial strains, including clinical isolates, but low toxicity compared to the original peptide. We envision that such modified versions of hECP30 may be suitable candidates for the design of new antimicrobial drugs.

## Results

### Rational Design of ECP Analogs and Stability in Serum

hECP30 (**1**, Table 1) is a 30-amino acid peptide with strong antimicrobial activity against Gram-negative bacteria, both in planktonic culture and biofilms.^29^ It includes six Arg residues, 20% of the total in its sequence. Arg and Lys are common targets for trypsin-like serine proteases.^30^ To avoid inactivation by protease cleavage, non-natural amino acid substitution is often used.^31^ In this case, replacement of all Arg residues in **1** by non-proteinogenic, cationic Orn, Dab, and Har improved stability in a moderate (∼2-fold) but significant way (Table 2).

**Table 1:** Primary Structures, HPLC Retention Times, and Molecular Mass of hECP30 Synthetic Analogs

				molecular mass (Da)	
entry	description	sequence^a^	HPLC retention time (min)^b^	theory	found
**1**	hECP30	RPFTRAQWFAIQHISPRTIAMRAINNYRWR	8.9	3757.4	3757.6
**2**	Orn analog	OPFTOAQWFAIQHISPOTIAMOAINNYOWO	8.4	3505.2	3505.2
**3**	Dab analog	XPFTXAQWFAIQHISPXTIAMXAINNYXWX	8.3	3420.9	3421.3
**4**	Har analog	ZPFTZAQWFAIQHISPZTIAMZAINNYZWZ	9.1	3841.5	3842.2
**5**	Des[Orn^1^-Pro^2^] analog	--FTOAQWFAIQHISPOTIAMOAINNYOWO	10.8	3293.9	3293.8
**6**	[Orn,^1^D-Pro^2^] analog	OpFTOAQWFAIQHISPOTIAMOAINNYOWO	10.9	3505.2	3505.2
**7**	[D-Orn,^1^D-Pro^2^] analog	opFTOAQWFAIQHISPOTIAMOAINNYOWO	10.8	3505.2	3505.2

a O, ornithine; X, 2,4-diaminobutyric acid; Z, homoarginine; d-amino acid residues in lower case.

b All peptides were analyzed by HPLC in a linear gradient of solvent B (acetonitrile) into solvent A (H_2_O) from 0 to 60% over 15 min.

**Table 2:** Peptide Half-Life Time in Incubations with 50% (v/v) Human Serum

peptide	half-life time (min)
**1**	12.3 ± 0.4
**2**	29.1 ± 0.5
**3**	20.6 ± 3.6
**4**	16.6 ± 0.5
**5**	>480
**6**	>480
**7**	>480

**Figure 2:**
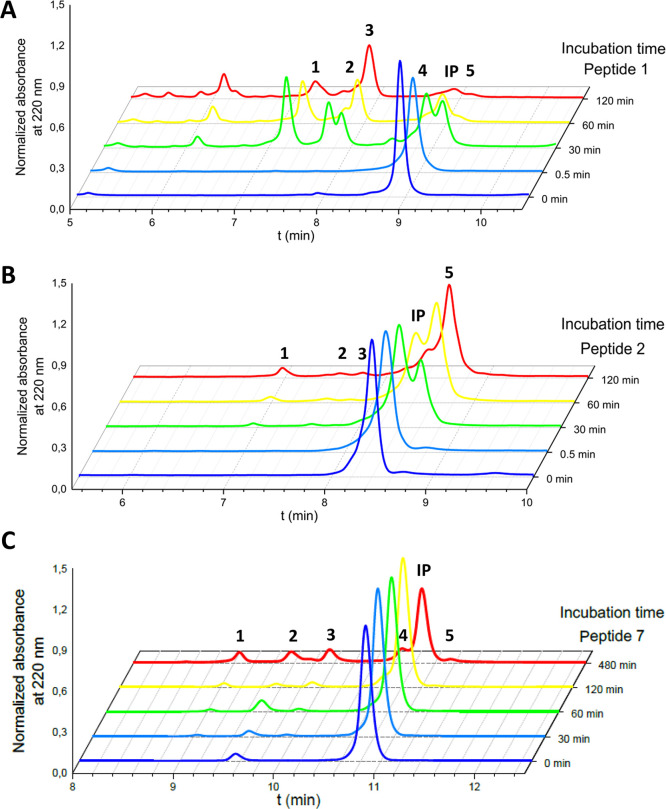
Peptide degradation profiles. 1 mM peptide stocks in water were incubated with human serum (1:1 v/v). Profiles shown are (A) hECP30 reference peptide **1**, (B) peptide **2**, and (C) peptide **7**. The amount of peptide remaining after incubation at different times was quantified by HPLC analysis. Peak numbers in each panel correspond to fragments listed on Table 3. IP: intact peptide.

The incubation profile of **1** with human serum shows extensive degradation, even at short times (Figure 2A). Although the three analogs are also rapidly degraded, they generate a second peak that is very stable over time, only further degraded at much longer incubation times (Figure 2 and Figures S1 and S2). These results suggest that Arg replacement protects all susceptible peptide bonds except one.

Most of the digestion subproducts could be identified by MS (Table 3), including the fragment with high stability (over 2 h), which was assigned to the original **1** peptide minus the first two N-terminal amino acids, Arg^1^-Pro^2^. To confirm this assignment, Des-OP (**5**, Table 1), a peptide lacking Arg(Orn)-Pro at the N-terminus and with Arg to Orn replacements at the other five positions, was prepared and tested, showing indeed a rather long half-life (>480 min). Prediction of the protease degradation profile using a bioinformatic tool (https://web.expasy.org/peptide_cutter/) identified proline endopeptidase as responsible for this fragment. On this basis, we argued that shielding of the Pro^2^-Phe^3^ peptide bond should prevent or minimize protease cleavage. To test this hypothesis, we synthesized two peptides where Pro^2^ was mutated to d-Pro and Orn was either in the l- or d-configuration (Op (**6**) and Op (**7**) analogs, respectively, Table 1). Both peptides displayed a strong improvement in stability, compared to **1** and **2**. The *t*_1/2_ > 480 min observed for peptides **6** and **7** represents a 30-fold stability increase over **1** (Table 2).

**Table 3:** Sequence of the Original Orn Analog Peptide **2** and Digestion Fragments Generated in Serum Incubations^a^

fragment	sequence
**1**	OPFTOAQWFAIQHISPO-------------
**2**	-----------------TIAMOAINNYOWO
**3**	OPFTOAQWFAIQHIS---------------
**4**	------QWFAIQHISPOTIAMOAINNY---
**5**	--FTOAQWFAIQHISPOTIAMOAINNYOWO
intact peptide	OPFTOAQWFAIQHISPOTIAMOAINNYOWO

a The fragments are classified in order of elution in HPLC analysis.

### Antimicrobial Activity

The antimicrobial activity of all peptides was measured and expressed as the minimal inhibitory concentration (MIC) against several bacterial strains. As ECP and **1** are most active against Gram-negative bacteria, MIC was determined against such bacterial species, including *Escherichia coli*, *Acinetobacter baumannii*, *Pseudomonas sp.*, *Salmonella enterica*, *Klebsiella pneumoniae*, and *Shigella flexneri.* The results confirmed the high antimicrobial activity of **1** against all Gram-negative strains (Table 4). Arg replacement with non-proteinogenic amino acids slightly reduced the activity, especially for **3** (Table 4). Analog **5**, which lacks the first two N-terminal residues, showed a marked reduction of activity against all strains, suggesting that these two residues are essential. Analog **7** had similar activity than the original peptide while **6** performed worse against some strains. Altogether, these results suggested that structural differences between **6** and **7** affect antimicrobial activity. All peptides show a minimal bactericidal concentration (MBC) within the same range as the MIC (Table S1). Results obtained by killing curve analysis for analogs **1**, **3**, **6**, and **7** also support these observations (Figure S3). In any event, most peptides display similar or slightly lower antimicrobial activity than LL-37, a well-known reference AMP (Table 4). When peptides were preincubated with human serum and tested against *Pseudomonas sp.*, we observed only a slight increase in the MIC values, suggesting that the peptide activity is barely affected by the presence of serum proteins (Table S2).

**Table 4:** MIC (μM) and Percentage of Hemolysis of hECP30 (**1**) and Analogs^a^

peptide	*E. coli*	*A. baumannii*	*Pseudomonas sp.*	*S. enterica*	*S. flexneri*	hemolysis % (250 μM peptide)	MRC-5 cytotoxicity (IC_50_, μM)
**1**	1.56	1.56	1.56	12.5	3.13	44 ± 4	23 ± 4
**2**	6.25	6.25	6.25	25	12.5	10 ± 2	113 ± 8
**3**	6.25	50	3.13	50	6.25	14 ± 6	182 ± 9
**4**	6.25	12.5	3.13	25	3.13	43 ± 1	7.6 ± 0.7
**5**	25	>100	25	>100	50	11 ± 4	> 300
**6**	3.13	12.5	1.56	50	50	1.1 ± 0.9	230 ± 40
**7**	3.13	6.25	1.56	25	6.25	3.6 ± 0.7	170 ± 20
LL-37	1.56	6.25	1.56	12.5	12.5	58 ± 7	64 ± 2

a LL-37 included for comparison.

In addition to the above reference strains, we also performed MIC assays on several clinical isolates. For some strains, i.e., *P. aeruginosa* and *S. typhimurium*, the concentrations required to inhibit bacterial growth were, on average, higher compared to the original peptide. Such behavior may be related to a higher net charge requirement to disrupt the cell membrane, as Orn is less basic than Arg.^32^ For *E. coli* and *A. baumannii*, MIC values for analogs were similar, or even better, than the reference peptide and, in general terms, **7** was substantially more active than **6** (Table 5). It is also worth noting that both hECP30 and its d-amino acid analogs show high activity against *A. baumannii* clinical isolates (Table 5), even at submicromolar concentrations. We also tested whether recurrent incubation of bacteria with peptides could induce resistance. To test for antibiotic resistance, we incubated *E. coli* cells for 7 rounds of evolution with concentrations below the MIC for the reference peptide **1** as well as analogs **6**, **7**, and LL-37 (1 μM) and ciprofloxacin (1 μM) as a positive control. We did not detect a significant increase in MIC for any of the compounds tested (Figure S4), whereas ciprofloxacin-treated cells increased their MIC from 1.5 to 5.0 μM after four cycles of incubation.

**Table 5:** MIC Values (μM) of hECP30 (**1**) and Analogs in Clinical Bacterial Strains

strain	peptide 1	peptide 6	peptide 7
*E. coli* 116878	6.25	25	6.25
*E. coli* cf073	3.13	6.25	3.13
*P. aeruginosa* 827651	25	25	100
*P. aeruginosa* 827632	12.5	>100	25
*S. typhimurium* 324	6.25	25	25
*S. typhimurium* 365	6.25	25	25
*A. baumannii* 3862	0.78	1.56	0.78
*A. baumannii* 3878	1.56	0.78	0.78
*A. baumannii* 3879	1.56	3.13	1.56
*A. baumannii* 3880	1.56	12.5	6.25

### Mechanism of Action

**Figure 3:**
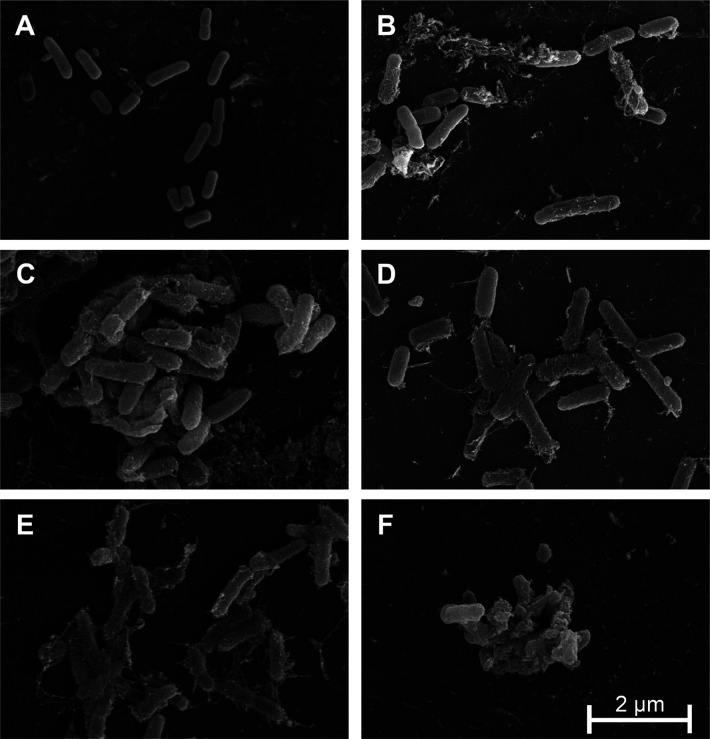
SEM images of *E. coli* in the mid-exponential phase (A) before treatment or after treatment with 10 μM of analogs (B) **1**, (C) **2**, (D) **6**, (E) **7**, or (F) LL-37.

The mechanism of action was elucidated by membrane depolarization and scanning electron microscopy (SEM). To study membrane depolarization, we used bacterial cells stained with the lipophilic dye DiSC_3_(5). In this assay, when membranes are compromised after peptide incubation, an increase in fluorescence is detected. Peptides **1**, **2**, **6**, and **7** displayed similar depolarization capacity although the kinetics was slightly slower for analog **6** (Figure S5). LL-37 was used as a positive control and showed a similar dye release, confirming the ability of all analogs to disrupt the bacterial cell membrane (Figure S5). Membrane damage was also studied by SEM in live bacteria. Peptide incubation showed in all cases a marked membrane damage, with blebs confirming that the structural integrity was compromised (Figure 3). The presence of blebs distributed all along the bacterial surface, the disruption of the cell morphology, and the presence of debris outside the cell suggest a carpet-like mechanism, as observed before for the ECP N-terminal domain,^21^ reinforcing the idea that the mechanisms of action of these analogs are similar.

### Toxicity Analysis

The antimicrobial activity must not be analyzed on its own but assessed alongside peptide toxicity. It is well known that unspecific and potent antimicrobial activity is often accompanied by high cytotoxicity against host cells.^33,34^ In our case, we tested cytotoxicity caused by the peptide by measuring lysis of horse red blood cells as monitored by hemoglobin release at 540 nm.^35^ The assay showed a high activity for reference peptide **1**, with ∼50% lysis at 250 μM, similar to LL-37 (Table 4). In contrast, **2** and **3** showed an important reduction in toxicity, with only 10% hemolysis at the same concentration (Table 4). For its part, the Har analog **4** showed lysis levels similar to **1** (42.86 ± 1.29%), suggesting that cytotoxicity is strongly related to cationic charge. Most interestingly, analogs **6** and **7** showed a massive reduction in cytotoxicity (1.12 ± 0.93 and 3.59 ± 0.69%, respectively), thus broadening the therapeutic window relative to the reference peptide.

The above results were compared to cytotoxicity assays in MRC-5 cells using the MTT assay. Results confirmed the high toxicity of peptides **1** and **4**, with IC_50_ values of 23 and 7.6 μM, respectively. In line with the hemolytic results, the toxicity of analogs **2** and **3** was markedly reduced, with IC_50_ values increased up to 180 μM (Table 4). No toxicity was detected for analog **5**, even at the highest concentration tested, concurring with its low antimicrobial activity. Finally, analogs **6** and **7** exhibited even lower toxicity than **5**, with IC_50_ values up to 230 μM. Such low toxicity, combined with a longer half-life, motivated us to further study these peptides from a structural point of view.

### Peptide Structure

A preliminary evaluation of the structure of peptides **1**, **2**, **6**, and **7** was provided by circular dichroism. Far UV spectra of peptides in aqueous solution or in the presence of SDS micelles (1 mM) were recorded. Spectra for all four peptides indicated a random conformation in aqueous solutions (Figure S6 and Table S3). Upon addition of SDS, the peptides adopted a more defined structure, mainly α-helical, in percentages ranging from 45 to 57%, consistent with previous results reported by our group for the ECP N-terminal domain (Table S3).^21^

**Figure 4:**
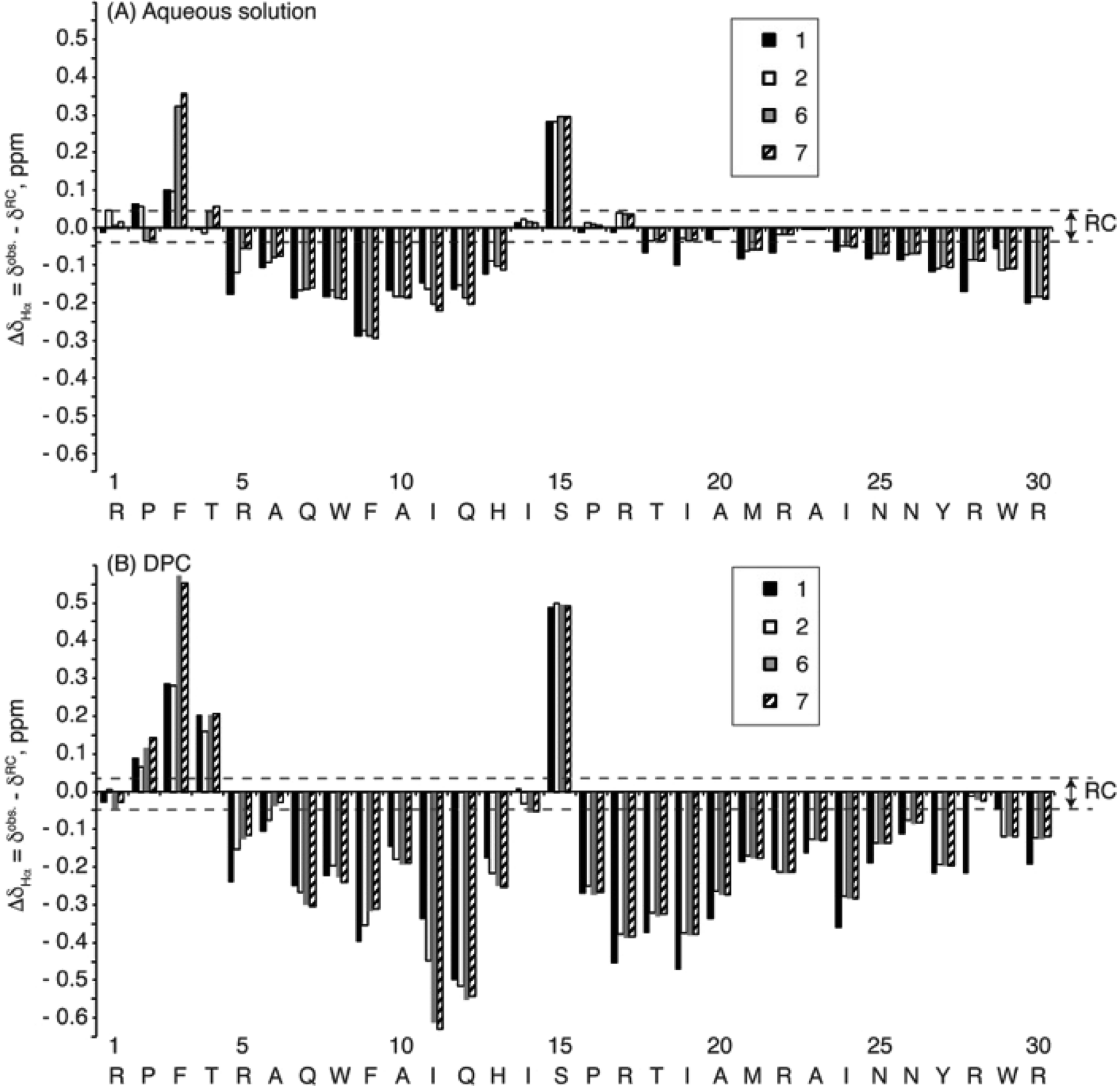
Bar plots of the Δδ_Hα_ values (Δδ_Hα_ = δ_Hα_^observed^ – δ_Hα_^RC^, ppm) as a function of sequence for peptides **1**, **2**, **6**, and **7** in (A) aqueous solution and (B) DPC micelles at pH 4.4 and 25 °C. Notice that the sequence of peptide **1** is shown and that Arg residues are Orn in peptides **2**, **6**, and **7**.

**Figure 5:**
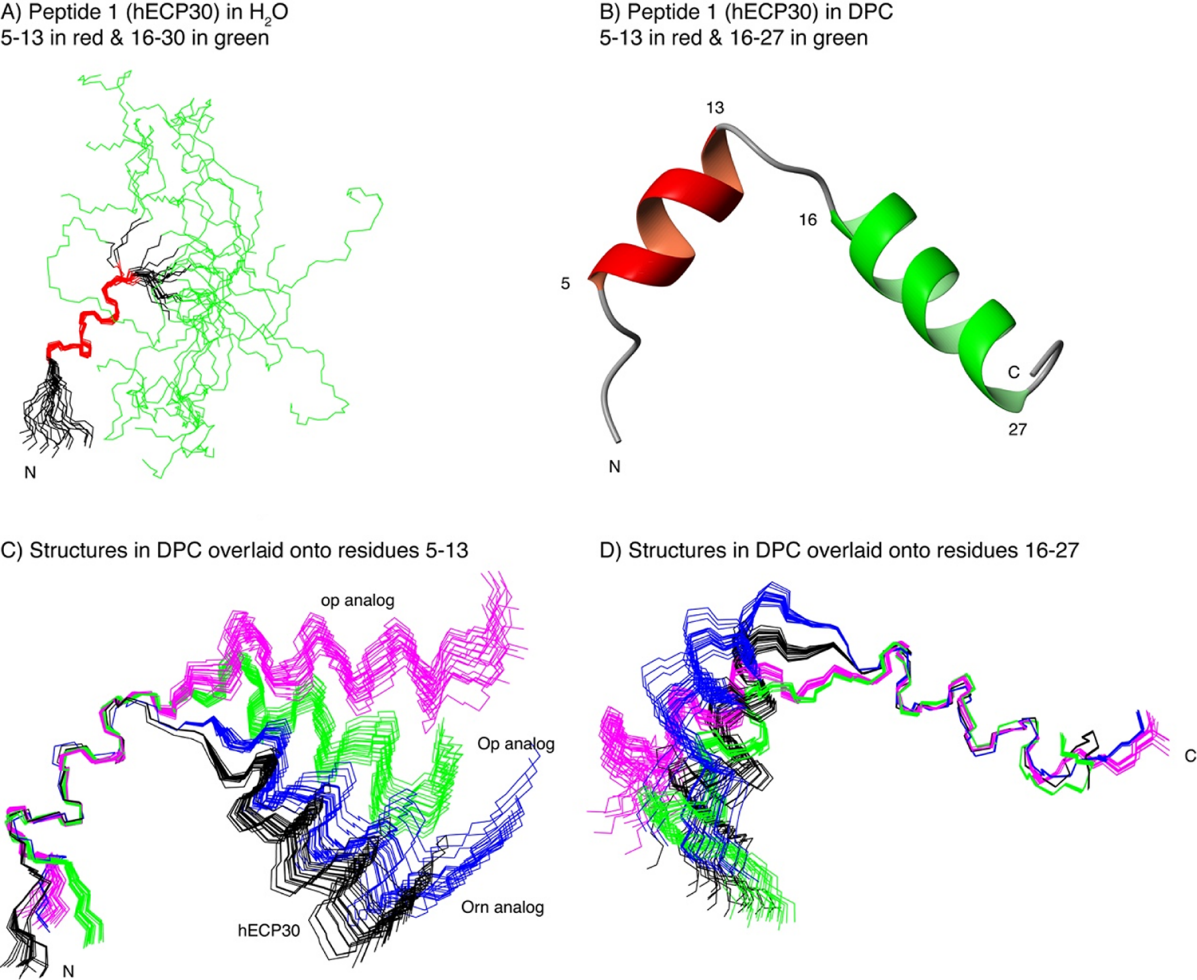
Peptide NMR calculated structures. (A) Ensemble of the 20 lowest target function structures of peptide **1** in aqueous solution overlaid onto residues 5–13. Residues 5–13 in red and 16–30 in green. (B) Ribbon representation of the lowest target function structure for peptide 1 in DPC micelles. (C, D) Ensembles of the 20 lowest target function NMR calculated structures for peptide **1** (black), **2** (blue), **6** (green), and **7** (magenta) analogs in DPC micelles overlaid onto residues 5–13 (panel C) and onto residue 16–27 (panel D).

**Figure 6:**
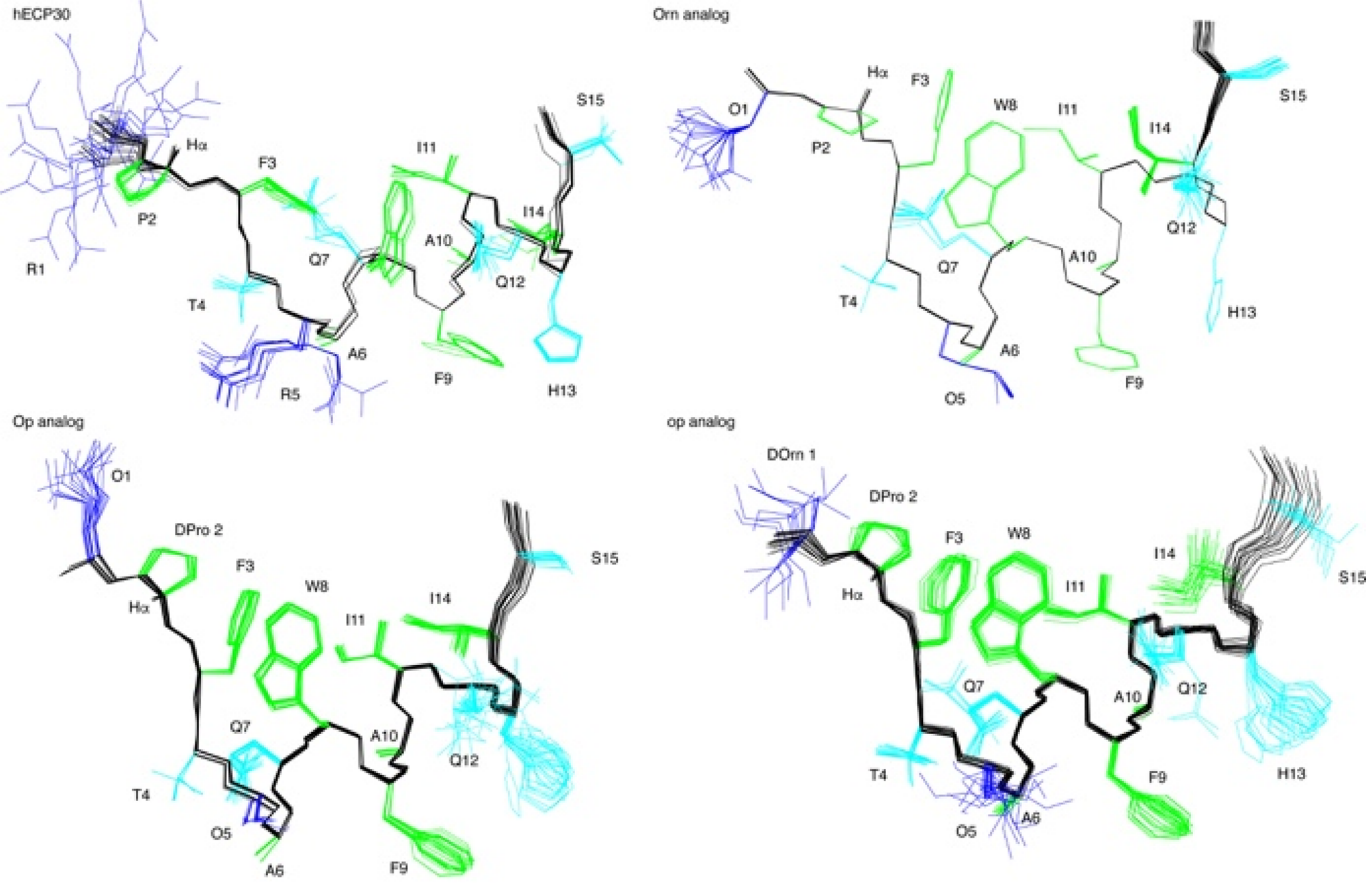
N-terminal regions (residues 1–15) of the NMR calculated structures for peptides **1**, **2**, **6**, and **7** in DPC micelles. Backbone atoms are displayed in black, Arg and Orn side chains in blue, polar side chains in cyan, and aliphatic and aromatic side chains in green. The H_α_ proton of Pro and d-Pro residues is shown.

Peptides **1**, **2**, **6**, and **7** were studied in aqueous solution and dodecylphosphocholine (DPC) micelles by NMR. All four peptides exhibited a helical segment spanning residues 5–13 and a slight helical tendency at residues 16–27 in aqueous solution, based on the analysis of the conformational shifts (Δδ_Hα_ and Δδ_Cα_; Figure 4, Table S4, and Figure S7). According to the magnitudes of the conformational shifts, both helices become more populated in the presence of DPC, in tune with the notion that micelles tend to stabilize amphipathic helices.^36,37^ On the whole, the peptides do not show any significant differences in secondary structure, i.e., helices span the same residues and there are no great differences in their populations (Table S4). More specifically, in aqueous solution, all four peptides show a well-defined N-terminal helix spanning residues 5–13, the only ones with non-sequential NOEs, and a mainly disordered C-terminal tail (Figure 5A, Figures S8 and S9, and Table S5). In contrast, in DPC micelles, the helix populations are higher and are better defined for the entire peptide length (Figures S8 and S9 and Table S5), with two helical regions spanning residues 5–13 and 16–27 (Figure 5B). A closer inspection reveals interesting differences among the four peptides, however, in the relative orientation of the helices (Figure 5C,D), and at the N-termini, where the d-residues in **6** and **7** also give rise to some variations. A detailed examination of side-chain packing at the N-terminal region (residues 1–15, including helix 5–13) was performed on the DPC structures, essentially identical to those in aqueous solution but better defined (Figure S7 and Table S5). In the four peptides, the helix exhibits a hydrophobic patch formed by residues F3, W8, I11, and I14 (Figure 6). The arrangement of these side chains seems to be slightly different in **1** compared to the other analogs. The hydrophobic patch extends to d-Pro2 in the case of **6** and **7**.

## Discussion and Conclusions

The replacement of original residues in peptides by non-proteinogenic or d-amino acids has been extensively studied over recent years to enhance AMP viability for clinical use.^38^ In AMPs, Arg and Lys are key cationic residues often involved in interactions with negatively charged bacterial membranes. However, these two residues are also a frequent target of serum proteases, with ensuing peptide inactivation. In hECP30, Arg represents 20% of the amino acid content; hence, we decided to investigate whether its replacement by non-coded surrogate residues could improve peptide stability and therapeutic potential.

We first observed that substituting Arg by Orn, Dab, or Har could moderately stabilize hECP30 against proteolytic cleavage in human serum (Table 2). This increase in half-life, up to 3-fold for analog **2**, may be explained by impaired recognition of Orn at the active site of proteases. Analyzing the degradation profile by HPLC–MS, we found an extremely stable cleavage byproduct in the modified analogs. This fragment had lost only the two residues (Orn and Pro) at the N-terminus; thus, we hypothesized that peptide **5** might be an interesting analog, largely impervious to proteolytic cleavage. Unfortunately, the improved stability was accompanied by low activity against bacterial strains, strongly suggesting that these first two residues are essential for antimicrobial action and cannot be omitted. Discarding therefore **5** as a candidate and analyzing the *in silico* peptide degradation profile by known serum proteases, we found that the Pro^2^–Phe^3^ bond was cleavable by a proline endopeptidase and hence decided to test two new analogs with either Pro^2^ or both Orn^1^ and Pro^2^ replaced by the corresponding d-enantiomers. As hypothesized, the new analogs exhibited remarkable serum stability, improving over 30-fold the half-life (>6 h vs 12 min for reference peptide **1**) and thus confirming that shielding the Pro^2^–Phe^3^ peptide bond from cleavage was crucial for stability. This is a remarkable improvement when compared to similar studies.^39^

Having improved stability, we next tested the impact of these changes on antimicrobial activity. We had detected a slight decrease in both analogs **2** and **3**, whereas the replacement of Arg by Har was neutral in terms of antimicrobial activity. These findings are readily explainable in structural terms: Har is very similar to Arg, both having a long side chain ending in a strongly cationic guanidine group, whereas Orn and Dab have shorter side chains ending in less basic amino functions (Figure 1). Importantly, shielding the Pro^2^–Phe^3^ bond by d-amino acid replacement in Orn analogs **6** and 7 improved both the half-life and the antimicrobial activity. Thus, MIC values for **7** were similar to the original peptide in almost all bacteria, both reference and clinical strains (Tables 4 and 5). These results suggest that bacteria may also use their own proteases to cleave peptides as a protective strategy, as observed before for other AMPs.^40^ Membrane depolarization assays and SEM images confirmed that the mechanism of action of the analogs is similar to the original ECP N-terminal domain, suggesting that the peptides would display a carpet-like mechanism.^21^ Also, no antimicrobial resistance was observed after cyclic incubations of bacteria with the peptides, suggesting that bacteria are less prone to developing resistance against these peptides compared to conventional antibiotics.

To measure the potential toxicity of the peptides, we performed assays to define *in vitro* therapeutic windows. Reference peptide **1** caused ∼50% hemolysis at 250 μM concentration (Table 4). Although this value may seem high, one must be reminded that it is about 100-fold the MIC in *E. coli*. Indeed, at lower concentrations, e.g., 10-fold the MIC, hemolytic activity is below 10%. On the other hand, while analogs **2** and **3** show a marked decrease in hemolytic activity, for **4**, the antimicrobial and hemolytic activities almost coincide, again suggesting that the chemical nature of the cationic residues is highly relevant for the mechanism of action. Thus, the strong cationic nature of Arg and Har increases antimicrobial activity of both peptides, by strongly binding the negatively charged bacterial membrane, whereas the long side chains also enhance, after charge neutralization, the hydrophobic nature of the residues, probably increasing hemolytic activity. Consistently with this hypothesis, Dab and Orn cause a marked reduction in hemolysis (<5% at 250 μM in both cases) and a slight decrease in activity which, upon N-terminal protease shielding (analogs **6** and **7**), rescues the antimicrobial potency of the original peptide.

To investigate whether the amino acid substitutions translated into peptide structure changes, we performed NMR analysis on the most promising candidates (**1**, **2**, **6**, and **7**) and found that the overall structure is conserved, with two main α-helices spanning residues 5–13 and 16–27 (Figures 3 and 4). The two helices are weakly formed in aqueous solution (30–40 and 10–15% for helices 5–13 and 16–27, respectively) but clearly defined in the presence of DPC micelles (70–80%). This is consistent with most AMPs and previous studies on the antimicrobial domain of ECP^23,41^ in contact with the negatively charged bacterial membrane. Even so, a slight conformational change in the second helix is observed in **2**, **6**, and **7** relative to **1**: the angle between the two helices increases upon replacing Arg by Orn, with peptides **2**, **6**, and **7** adopting a more extended conformation that may help explain the slightly lower activity of Orn analogs compared to the original peptide.

In light of the present results, we conclude that peptide **7** is a promising AMP lead. Its high antimicrobial activity and low cytotoxicity portray it as an excellent candidate to fight infections caused by Gram-negative bacteria. Moreover, its remarkable in vitro serum stability suggests that it would survive in the organism for a prolonged time.

## Experimental Section

### Materials

Purified horse red blood cells were acquired from Thermo Fisher Scientific (Hampshire, England). Human serum was purchased from Sigma (St Louis, USA). *E. coli* was obtained from the Coli Genetic Stock Center (BW25113). *S. flexneri* (ATCC 29903), *A. baumannii* (ATCC 15308), *Pseudomonas sp.* (ATCC 15915), and *S. enterica* (ATCC 14028) were obtained from the CECT (Valencia, Spain). Clinical strains were supplied by the Vall d’Hebron Hospital (Barcelona, Spain). MRC5 fibroblasts were obtained from the Cytometry and Cell Culture Facility (SCAC, UAB) and originally purchased from ATCC.

### Peptide Synthesis

Peptides were assembled in the C-terminal carboxamide form at a 0.1 mmol scale on a H-Rink Amide-ChemMatrix resin of 0.50 mmol/g substitution (PCAS BioMatrix, Quebec, Canada) in a Prelude instrument (Gyros Protein Technologies, Tucson, AZ) using Fmoc solid-phase peptide synthesis (SPPS) protocols. After chain assembly, peptides were fully deprotected and cleaved from the resin with TFA/H_2_O/triisopropylsilane (95:2.5:2.5 v/v) for 90 min at RT with gentle agitation. Peptides were precipitated from the TFA solution by addition of chilled diethyl ether followed by three centrifugations at 4800 rpm, 5 min, 4 °C, taken up in water and lyophilized. Crude peptides were checked by analytical RP-HPLC and LC–MS and purified by preparative RP-HPLC. Analytical RP-HPLC was performed on an LC-20 AD instrument (Shimadzu, Kyoto, Japan) fitted with a Luna C18 column (4.6 mm × 50 mm, 3 μm; Phenomenex) using linear gradients of solvent B (0.036% TFA in ACN) into A (0.045% TFA in H_2_O) over 15 min, at 1 mL/min flow rate and with UV detection at 220 nm. Preparative RP-HPLC was performed on an LC-8 instrument (Shimadzu) fitted with a Luna C18 column (21.2 mm × 250 mm, 10 μm; Phenomenex), using linear gradients of solvent B (0.1% TFA in ACN) into A (0.1% TFA in H_2_O) over 30 min, with a flow rate of 25 mL/min. MS analysis was performed on an LC–MS 2010EV instrument (Shimadzu) fitted with an Aeris Widepore XB-C18 column (150 × 4.6 mm, 3.6 μm, Phenomenex), eluting with linear gradients of B (0.08% formic acid (FA) in ACN) into A (0.1% FA in H_2_O) over 15 min at a 1 mL/min flow rate. Fractions of >95% HPLC purity and with the expected mass by LC–MS were pooled and lyophilized. Peptide purity was assessed by the area of the purified peptide peak relative to the total peak areas in the chromatogram. Peptide stock solutions were prepared in sterile deionized water and stored at −20 °C.

### Peptide Stability

To determine stability, 1 mM peptide stocks were incubated with human serum in a 1:1 ratio. At various incubation times, aliquots were removed, and digestion was stopped by ACN precipitation. After discarding serum proteins by centrifugation, original peptides and proteolysis fragments in the supernatant were analyzed by LC–MS. Solvents and methods are as above. Results are the average of three independent studies.

### Minimum Inhibitory Concentration (MIC) and Minimum Bactericidal Concentration (MBC)

The antibiotic activity was defined by the minimum peptide concentration where the microorganism is unable to grow. The assay was done in polypropylene 96-well plates (Greiner, Frickenhausen, Germany) to avoid peptide binding to plate wells. Peptides were dissolved in water containing 0.4% w/v bovine serum albumin (BSA) and 0.02% v/v glacial acetic acid to prevent self-aggregation, following the reference protocol by Wiegand et al.,^42^ based on the classical microtiter broth dilution recommended by the National Committee of Laboratory Safety and Standards (NCLSS). Initial bacteria inoculum was adjusted to 5 × 10^5^ CFU/mL in Mueller-Hinton (MH) medium. Incubations were kept at 37 °C for 24 h. Results are the average of three independent studies. MIC assays in the presence of serum were performed by incubating the peptide with human serum at a 1:1 ratio. Then, 10^6^ cfu/mL of *Pseudomonas sp.* were incubated with serial dilutions of the peptides for 4 h. To determine the MBC, 50 μL from all wells were seeded into Petri plates and incubated for 24 h. The MBC concentration was attributed to the lowest concentration without detectable bacterial growth for each peptide.

### Antimicrobial Resistance

To test whether recurrent incubation of bacteria with peptides could induce resistance, *E. coli* (10^7^ cfu/mL) were incubated with peptides (1 μM) or ciprofloxacin (1 μg/mL) for 1 h. After incubation, cells were centrifuged, washed, and grown overnight in Mueller-Hinton medium. The cycle of incubation and growth was repeated in a cyclic manner for 7 days, and each day, cultures were tested for MIC to detect resistance.

### Killing Curve Assay

Killing curves were followed using the live/dead bacterial viability kit (Invitrogen, Oregon, USA). Briefly, fresh bacteria cultures (*E. coli*, *A. baumannii*, and *Pseudomonas sp.*) were brought to a concentration of approximately 4 × 10^8^ cfu/mL for the assay. In a polypropylene 96-well plate (Greiner, Frickenhausen, Germany), 100 μL of a 3:2 mixture of bacteria and live/dead reagent in phosphate-buffered saline (PBS), incubated previously for 15 min, was added to each well containing 50 μL of 1:2 serially diluted peptide in PBS (concentration range from 100 μM to 0.2 μM). The fluorescence emission was continuously recorded for 12 h with a Tecan Infinite F Nano+ microplate reader (Tecan, Germany) using an excitation wavelength of 485 nm. Emission wavelength for SYTO9 was set to 530 nm (green, living cells) and 630 nm for propidium iodide (red, dead cells). Results are the average of three independent studies.

### Hemolytic Activity

The peptide toxicity was determined detecting the disruption of horse red blood cells, following a previous assay report.^35^ 10-fold diluted cells in PBS were centrifuged three times at 800*g* to eliminate the hemoglobin from disrupted cells. After the last resuspension, erythrocytes were incubated with the peptides for 4 h at 37 °C. The detection was done measuring the supernatant absorbance in a VICTOR3 multilabel plate reader (Perkin Elmer, Waltham, Massachusetts) at 540 nm in a microplate reader. Percentage of hemolysis was calculated through a pattern done with dilutions of total cell lysis done with a 1% Triton X-100 buffer. Results are the average of three independent studies.

### Bacterial Membrane Depolarization

Membrane depolarization on *E. coli* was performed by the quenching and later release of DiSC_3_(5) (Fisher, Hampshire, England) as described in Zhang et al., with some modifications.^43^*E. coli* fresh cultures were brought to exponential growth (0.2–0.4 OD), washed twice with 5 mM HEPES, 20 mM glucose, and 100 mM KCl, pH 7.2, and finally resuspended with the same buffer up to an OD of 0.05. Samples were mixed with the dye to a final concentration of 0.4 μM and incubated in darkness from 20 to 30 min to obtain a stable baseline. The peptides were added to the mixture and DiSC_3_(5) release was measured in a Varian Cary Eclipse fluorescence spectrometer (Agilent, Santa Clara, California) with excitation and emission at 625 and 666nm and 5 nm and 10 nm excitation and emission slit, respectively.

### Scanning Electron Microscopy (SEM)

*E. coli* cell cultures were grown in LB at 37 °C to the mid-exponential phase (OD_600_ = 0.4). 1 mL of cell culture was incubated for 2 h with 10 μM peptides at room temperature. Samples were then prepared for analysis, as described previously.^44^ Briefly, the cell suspensions were filtered through 0.1 μm Nucleopore filters to retain the bacteria and then fixed with 2.5% glutaraldehyde in 100 mm Na-cacodylate buffer (pH 7.4) for 2 h at 4 °C. Attached cells were post-fixed by immersing the filters in 1% osmium tetroxide in Na-cacodylate buffer for 30 min, rinsed in the same buffer, and dehydrated in ethanol in ascending percentage concentrations [31, 70, 90 (×2), and 100 (×2)] for 15 min each. The filters were mounted on aluminum stubs and coated with gold–palladium in a sputter coater (K550; Emitech, East Grinsted, UK). The filters were viewed at 15 kV accelerating voltage in a EVO MA 10 scanning electron microscope (Zeiss, Oberkochen, Germany).

### Cytotoxicity in Mammalian Cells

MRC-5 cells were grown in Eagle’s minimum essential medium (MEMα), supplemented with 10% fetal bovine serum (FBS). 96-well plates were seeded with 3 × 10^4^ cells/well and cultured overnight in order to get cells adhered into the plate and then incubated with two-fold peptide serial dilutions. After 4 h of incubation, the medium containing the peptides was replaced by MEMα supplemented with FBS and 3-(4,5-dimethylthiazol-2-yl)-2,5-diphenyltetrazolium bromide (MTT) at 0.4 mg/mL and incubated for 150 min. Detection of formazan crystals in the living cells was done by cell disruption by addition of 200 μL of dimethyl sulfoxide (DMSO) followed by absorbance measuring at 600 nm in a Tecan Infinite F Nano+ microplate reader (Tecan, Germany). Results are the average of three independent studies.

### Circular Dichroism

The CD spectrum of peptides **1**, **2**, **6**, and **7** was measured in 5 mM sodium phosphate pH 7 or 5 mM sodium phosphate buffer pH 7 with 1 mM SDS to simulate a membrane environment. Peptide final concentration was 10 μM. Samples were analyzed in a Jasco J-815 CD spectropolarimeter (Jasco, Easton, Maryland) in 0.2 mm quartz cuvettes (Hellma, Germany). Far UV spectra were measured from 260 to 190 nm and analyzed using the CDSSTR method to predict the secondary structure.^45^

### NMR Spectroscopy

NMR samples were prepared by dissolving the lyophilized peptides at about 1 mM concentration in aqueous solution (H_2_O/D_2_O 9:1 v/v or pure D_2_O) or in DPC micelles (50 mM [D_38_]-DPC in H_2_O/D_2_O 9:1, v/v or in pure D_2_O). The pH was measured using a glass microelectrode and adjusted to 4.4 by addition of NaOD or DCl. Sodium 2,2-dimethyl-2-silapentane-5-sulfonate (DSS) at a 0.1–0.2 mM concentration was added as internal reference for the ^1^H chemical shifts.

As previously reported,^46^ a Bruker Avance-600 spectrometer equipped with a cryoprobe was used to record NMR spectra: 1D ^1^H, 2D ^1^H,^1^H-COSY (phase-sensitive two-dimensional correlated spectroscopy), ^1^H,^1^H-TOCSY (total correlated spectroscopy), ^1^H,^1^H-NOESY (nuclear Overhauser enhancement spectroscopy), and ^1^H-^13^C-HSQC (heteronuclear single quantum coherence) at ^13^C natural abundance. TOCSY and NOESY mixing times were 60 and 150 ms, respectively. Data were processed using the TOPSPIN software (Bruker Biospin, Karlsruhe, Germany).

SPARKY software^47^ was used to analyze the NMR spectra. The ^1^H chemical shifts were assigned by analysis of the 2D homonuclear spectra using the well-established sequential assignment methodology,^48^ and the ^1^H-^13^C-HSQC spectra were analyzed to assign the ^13^C chemical shifts. The assigned chemical shifts have been deposited at the BioMagResBank (http://www.bmrb.wisc.edu) with accession codes BMRB ID: 50486-50493.

The conformational shifts for Hα protons (Δδ_Hα_, ppm) and Cα carbons (Δδ_Cα_, ppm) were obtained using the following equations: Δδ_Hα_ = δ_Hα_^observed^ – δ_Hα_^RC^, ppm and ΔδCα = δ_Cα_^observed^ – δ_Cα_^RC^, ppm; where δ_Hα_^observed^ and δCα^observed^ are, respectively, the Hα and Cα chemical shifts observed for the peptides, and the random coil values δ_Hα_^RC^ and δ_Cα_^RC^ were taken from ref (49).

Helix populations were estimated from the ^1^H_α_ and ^13^C_α_ chemical shifts as previously described.^46^

Peptide structures were calculated using the iterative procedure for automatic NOE assignment integrated in the CYANA 3.98 program.^50^ This algorithm consists of seven cycles of combined automated NOE assignment and structure calculation, in which 100 conformers were computed per cycle. The experimental input data comprises the lists of assigned chemical shifts, and NOE integrated cross-peaks present in 150 ms NOESY spectra, plus the ϕ and ψ dihedral angle restraints. The NOE cross-peaks were integrated using the automatic integration subroutine of the SPARKY software.^47^ The TALOSn webserver^51^ was used to obtain the dihedral angle restraints from the^1^H and^13^C chemical shifts. The final structure of each peptide is the ensemble of the 20 lowest target function conformers calculated in the last cycle. These ensembles were visualized and examined using the MOLMOL program.^52^ The coordinates for the calculated structures are available upon request from the authors.

## Supporting Information Available

Peak integration data for all peptides after incubation with human serum; degradation profiles for peptides **3** and **4**; *A. baumannii* survival ratio after incubation with peptides; antimicrobial resistance; membrane depolarization after incubation with peptides; circular dichroism spectra for peptides in the presence and absence of SDS micelles; Δδ_Cα_ values for peptides **1**, **2**, **6**, and **7** in aqueous solution and DPC micelles; NMR solution structures for peptides **1**, **2**, **6**, and **7**; NMR solution structures for C-Terminal regions of peptides **1**, **2**, **6**, and **7**; MBC values; antimicrobial activity in the presence of human serum for peptides **1**, **2**, **3**, and **4**; predicted secondary structure of peptides; averaged Δδ_Hα_ and Δδ_Cα_ values for peptides **1**, **2**, **6**, and **7** in aqueous solution and DPC micelles; structural statistics parameters for peptide structures **1**, **2**, **6**, and **7**; HPLC traces and MS spectra for all peptides (PDF)
Molecular formula strings (CSV)

## ABBREVIATIONS

AMP: antimicrobial peptide
ECP: eosinophil cationic protein
ACN: acetonitrile
MIC: minimum inhibitory concentration
Orn: ornithine
Dab: 2,4-diaminobutyric acid
Har: homoarginine
PBS: phosphate buffered saline
MTT: 3-(4,5-dimethylthiazol-2-yl)-2,5-diphenyltetrazolium bromide
