# Supplementary material for: Rationally Modified Antimicrobial Peptides from the N-Terminal Domain of Human RNase 3 Show Exceptional Serum Stability
Source: J Med Chem. 2021 Aug 3;64(15):11472–82. doi: 10.1021/acs.jmedchem.1c00795 (PMC8483441; doi:10.1021/acs.jmedchem.1c00795)
Supplement: Supplementary file 1 — jm1c00795_si_001.pdf [file jm1c00795_si_001.pdf]

SUPPORTING INFORMATION

for

Rationally modified antimicrobial peptides from the N-terminal domain of human RNase3 show exceptional serum stability

Daniel Sandín[1], Javier Valle[2], Belén Chaves-Arquero[3], Guillem Prats-Ejarque[1], María Nieves Larrosa[4], Juan José González[4], María Ángeles Jiménez[3], Ester Boix[1,*], David Andreu[2,*] and Marc Torrent[1,*]

[1] Department of Biochemistry and Molecular Biology, Universitat Autònoma de Barcelona, 08193, Cerdanyola del Vallès, Spain.

[2] Department of Experimental and Health Sciences, Universitat Pompeu Fabra, Barcelona Biomedical Research Park, 08003 Barcelona, Spain.

[3] Departamento de Química-Física Biológica, Instituto de Química Física Rocasolano (IQFR-CSIC), Serrano 119, 28006, Madrid, Spain.

[4] Servei de Microbiologia, Hospital Vall d'Hebron, 08035, Barcelona, Spain.

Correspondence to: Ester Boix: ester.boix@uab.cat; David Andreu: david.andreu@upf.edu; Marc Torrent: marc.torrent@uab.cat.

TABLE OF CONTENTS

Title page	Page S-1
Supplementary Figure 1	Page S-2
Supplementary Figure 2	Page S-3
Supplementary Figure 3	Page S-4
Supplementary Figure 4	Page S-5
Supplementary Figure 5	Page S-6
Supplementary Figure 6	Page S-7
Supplementary Figure 7	Page S-8
Supplementary Figure 8	Page S-9
Supplementary Figure 9	Page S-10
Supplementary Table 1	Page S-11
Supplementary Table 2	Page S-12
Supplementary Table 3	Page S-13
Supplementary Table 4	Page S-14
Supplementary Table 5	Page S-15
Supplementary Material	Pages S-16 - S-25

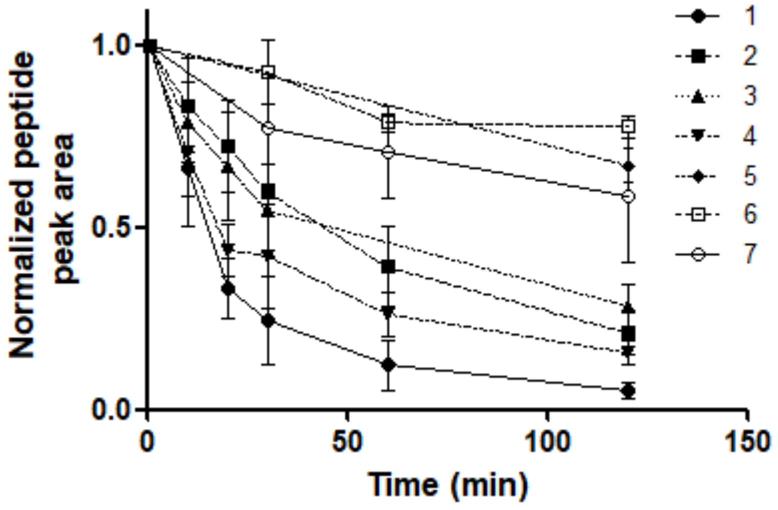

Supplementary Figure 1. Normalized peak area of the remaining intact peptide after incubation with human serum at different time points for all analogs synthesized.

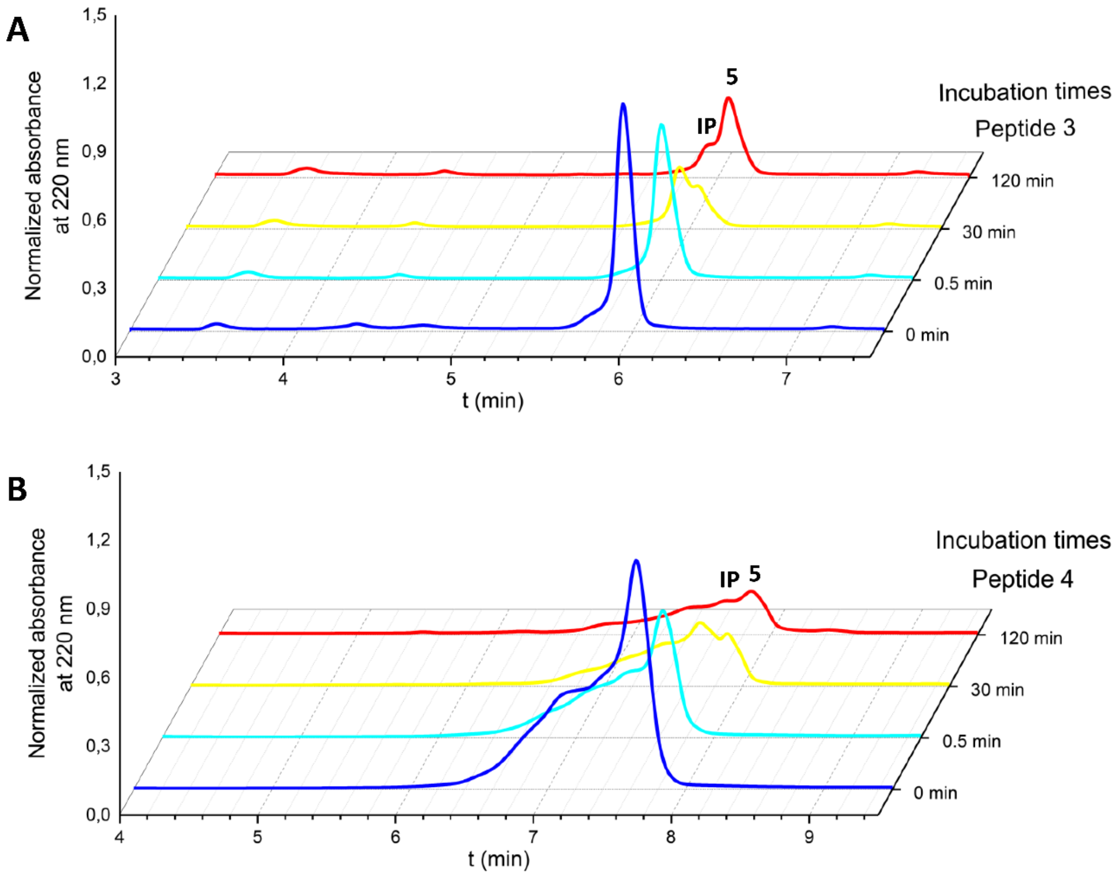

Supplementary Figure 2. Peptide degradation profiles obtained by incubation of peptides at 1mM concentration in water with human serum in a 1:1 ratio. Panel (A) Dab analog **3** and (B) Har analog **4**. Quantitation of remaining peptide at different incubation times was done by HPLC analysis. The numbers in each plot correspond to the fragments listed in Table 3. CP is the complete peptide.

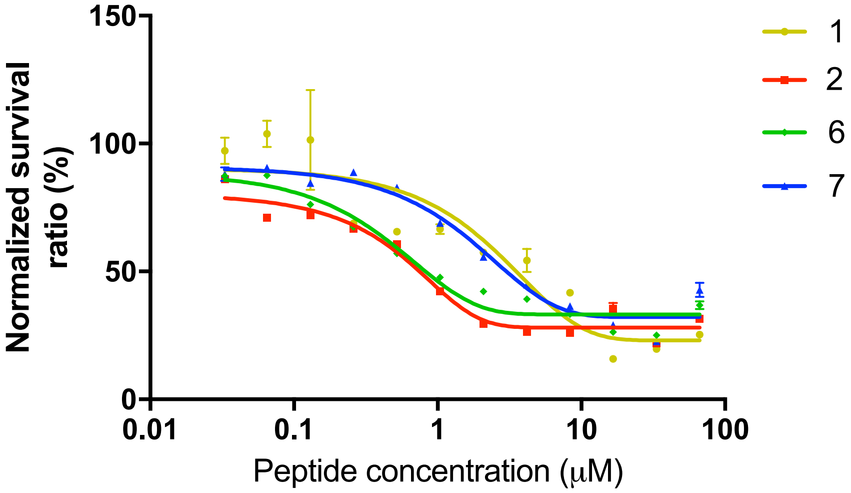

Supplementary Figure 3. Bacterial survival ratio after incubation of *A. baumannii* cells for 2 h with increasing peptide concentrations. Survival ratio was evaluated as the syto9/IP fluorescence ratio and normalized using unchallenged bacteria cells (100% survival) and dead cells (5% triton X-100, 0% survival).

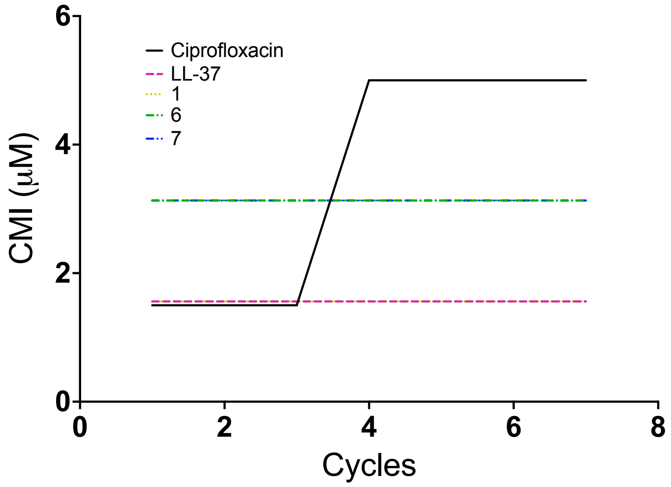

Supplementary Figure 4. Antimicrobial resistance against *E. coli* for all peptides tested. Cells were treated with antibiotics or peptides for 7 cycles and the MIC was measured after each incubation cycle.

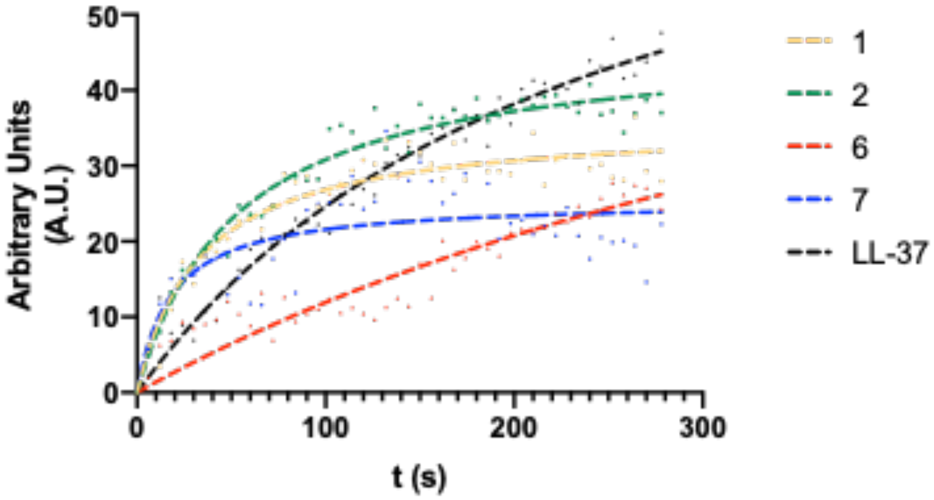

Supplementary Figure 5. Detection of DiSC$_3$(5) lipophilic dye release due to depolarization of the bacterial membrane

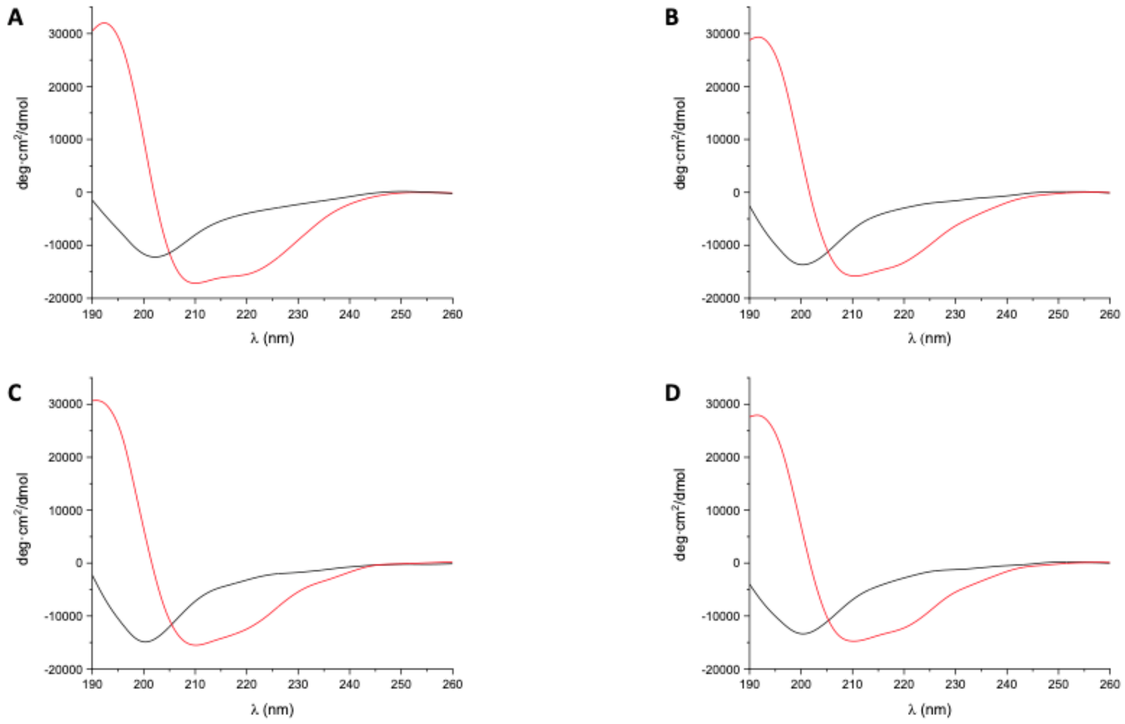

Supplementary Figure 6. Circular dichroism spectra from 260 to 190 nm of (**A**) peptide **1**, (**B**) peptide **2**, (**C**) peptide **6** and (**D**) peptide **7** in the presence of 5mM PBS (black lines) or 5mM PBS and 1 mM SDS (red lines).

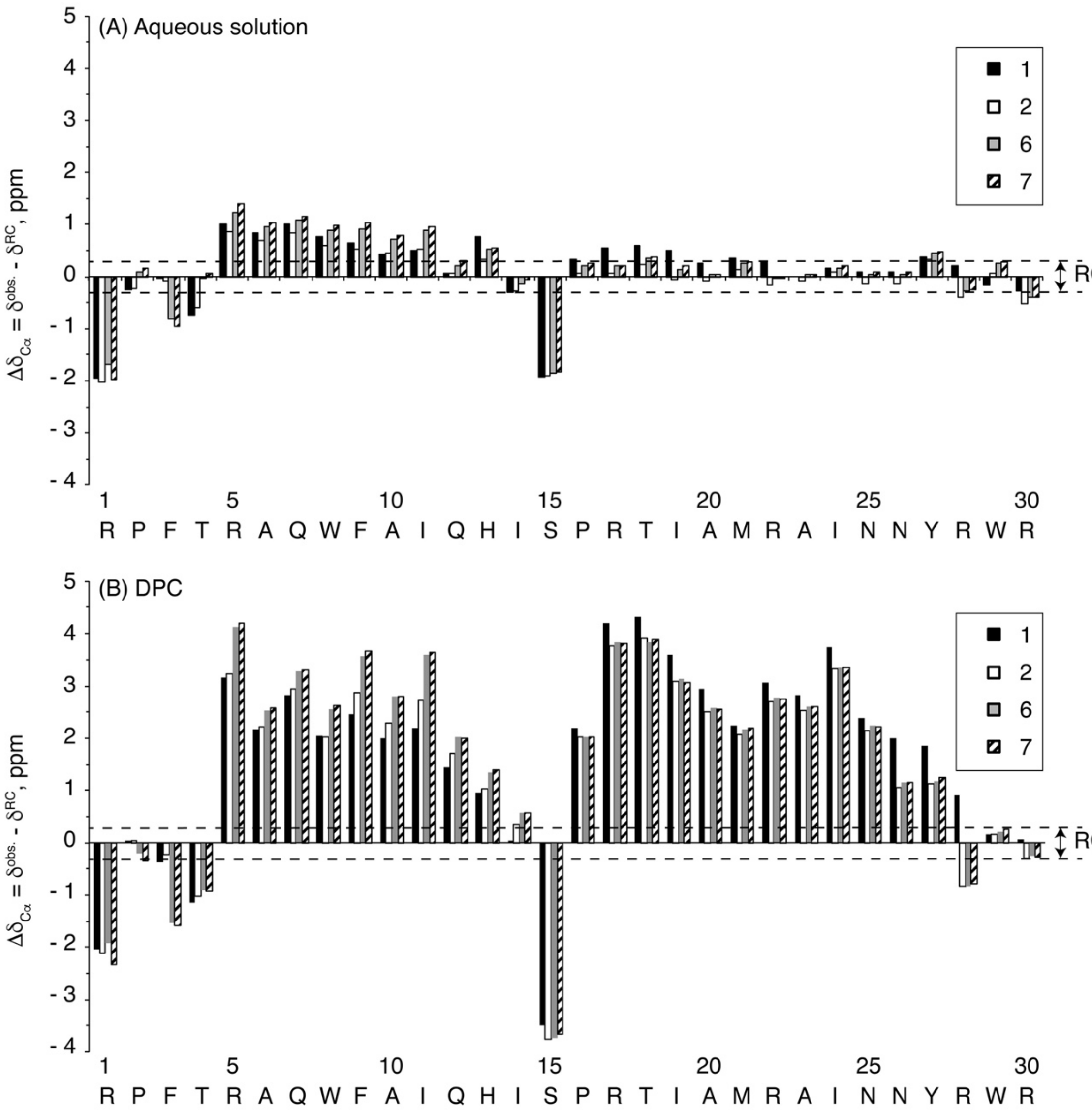

Supplementary Figure 7. Bar plots of the $\Delta\delta_{C\alpha}$ values ($\Delta\delta_{C\alpha} = \delta_{C\alpha}^{observed} - \delta_{C\alpha}^{RC}$, ppm) as a function of sequence for peptides **1**, **2**, **6** and **7** in aqueous solution (A) and in DPC micelles (B) at pH 4.4 and 25ºC. $\delta_{H\alpha RC}$ were taken from Wishart et al. 1995. Notice that the sequence of peptide **1** is shown, and that Arg residues are Orn in peptides **2**, **6** and **7**.

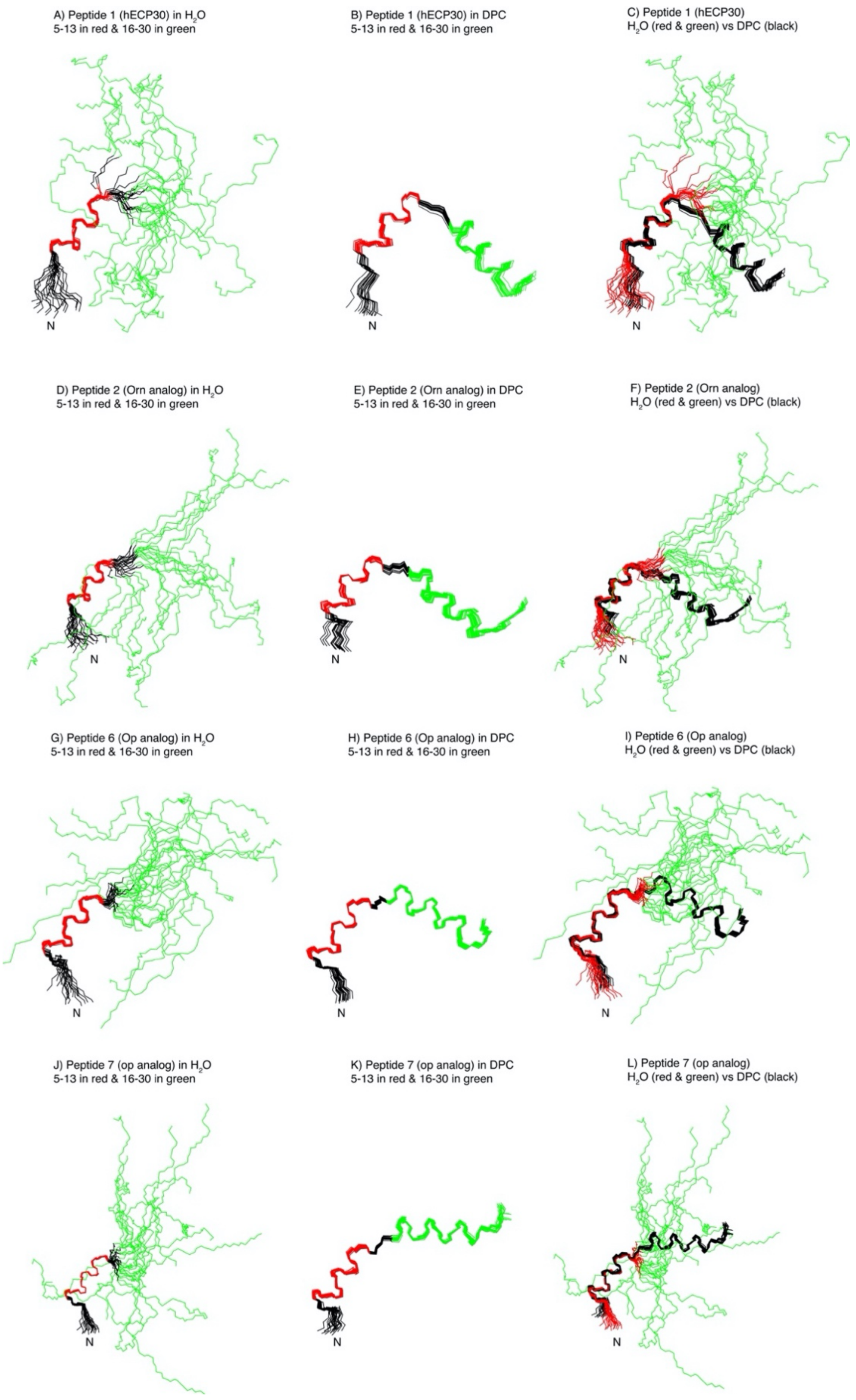

Supplementary Figure 8. NMR solution structures for peptides **1**, **2**, **6** and **7** in aqueous solution (A, D, G & J) and in DPC terminal regions (B, E, H & K). An overlay of the structures in aqueous solution (red and green) and in DPC (black) are shown in panels C, F, I and L.

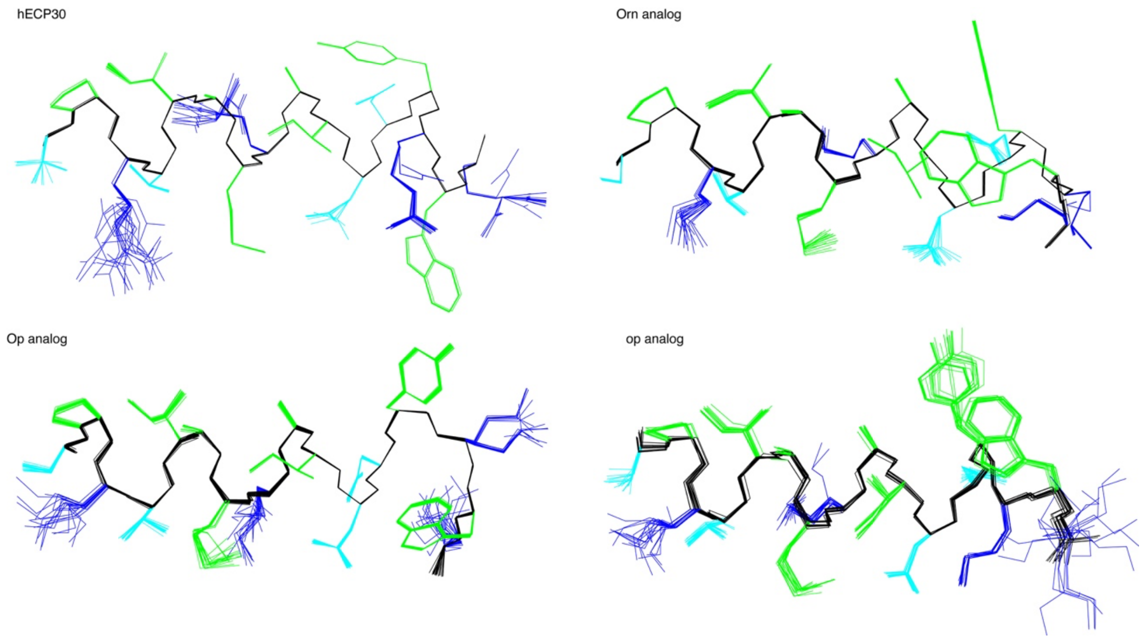

Supplementary Figure 9. C-terminal regions (residues 15-30) of the NMR calculated structures for peptides **1**, **2**, **6** and **7** in DPC micelles. Backbone atoms are displayed in black, Arg and Orn side chains in blue, polar side chains in cyan and aliphatic and aromatic side chains in green.

Supplementary Table 1. MBC (μM) of the peptides tested.

Peptide	*Escherichia coli*	*Acinetobacter baumannii*	*Pseudomonas sp.*	*Salmonella enterica*	*Shigella flexneri*
**1**	1.56	1.56	1.56	12.5	3.13
**2**	6.25	6.25	6.25	25	12.5
**3**	6.25	50	3.13	50	6.25
**4**	6.25	12.5	3.13	25	3.13
**5**	25	>100	25	>100	50
**6**	3.13	12.5	1.56	50	50
**7**	3.13	6.25	1.56	25	6.25
**LL-37**	1.56	6.25	1.56	12.5	12.5

Supplementary Table 2. Antimicrobial activity in *Pseudomonas sp.* for peptides **1**, **2**, **3** and **4 incubated with human serum.**

Peptide	MIC (µM)
**1**	25
**2**	50
**3**	12.5
**4**	6.25

Supplementary Table 3. Predicted secondary structure for peptides **1**, **2**, **6** and **7** according to circular dichroism.

Peptide	α-Helix	β-Strand	Turns	Unordered
**1 (PBS)**	0.12	0.30	0.26	0.32
**1 (PBS + SDS)**	0.57	0.10	0.13	0.23
**2 (PBS)**	0.10	0.30	0.25	0.35
**2 (PBS + SDS)**	0.49	0.14	0.13	0.24
**6 (PBS)**	0.11	0.30	0.25	0.34
**6 (PBS + SDS)**	0.46	0.16	0.14	0.24
**7 (PBS)**	0.09	0.32	0.25	0.34
**7 (PBS + SDS)**	0.47	0.15	0.13	0.25

Supplementary Table 4. Averaged $\Delta\delta_{H\alpha}$ and $\Delta\delta_{C\alpha}$ values for the peptides in aqueous solution and in the presence of DPC micelles (50 mM DPC) at pH 4.4 and 25 ºC. Percentage of helical structure was estimated from these values.

Aqueous solution						
Peptide	Helical residues	$\Delta\delta_{H\alpha}$, ppm	% helix	$\Delta\delta_{C\alpha}$, ppm	% helix	Av. % helix[a]
**1**	5-13	-0.171	44	0.67	22	33±11
	16-27	-0.063	16	0.31	10	13±3
**2**	5-13	-0.156	40	0.55	18	29±11
	16-27	-0.045	12	0.15	5	9±4
**6**	5-13	-0.167	43	0.91	30	37±7
	16-27	-0.045	12	0.21	7	10±3
**7**	5-13	-0.161	41	0.82	27	34±7
	16-27	-0.044	11	0.16	5	8±3
DPC micelles						
**1**	5-13	-0.264	68	2.13	69	69±1
	16-27	-0.274	70	2.94	95	83±13
**2**	5-13	-0.266	68	2.33	75	72±4
	16-27	-0.231	59	2.52	82	71±12
**6**	5-13	-0.290	71	2.91	94	83±12
	16-27	-0.237	61	2.57	83	72±11
**7**	5-13	-0.291	71	2.87	93	82±11
	16-27	-0.239	61	2.57	83	72±11

[a] Errors are reported as the standard deviation.

Supplementary Table 5. Summary of structural statistics parameters for the ensemble of the 20 lowest target function conformers calculated for peptides in aqueous solution and in DPC micelles.

**Peptide**	**1**	**2**	**6**	**7**	**1**	**2**	**6**	**7**
**Conditions**	**Aqueous solution**				**DPC micelles**			
**Number of distance restraints**								
Intraresidue & sequential (i – j ≤ 1)	132	141	192	199	305	281	282	261
Medium range (1 < |i – j| < 5)	7	6	21	21	160	166	150	143
Long range (|i – j| ≥ 5)	0	0	5	5	15	28	20	19
Total number	139	147	218	225	480	475	452	423
Averaged total number per residue	4.6	4.9	7.3	7.5	16	15.8	15.1	14.1
**Number of dihedral angle constraints**								
Number of restricted ϕ angles	26	26	26	26	26	25	26	26
Number of restricted ψ angles	15	17	18	17	27	24	26	27
Total number	41	43	44	43	53	49	52	53
**Pairwise RMSD (Å)**								
**Residues 1-30**								
Backbone atoms	7.9±2.5	9.0±3.1	7.7±2.5	8.3±2.5	0.6±0.3	0.6±0.4	0.6±0.2	0.4±0.2
All heavy atoms	9.9±2.5	10.1±3.0	9.0±2.6	9.3±2.5	1.3±0.3	0.8±0.4	1.1±0.2	0.8±0.1
**Residues 5-13 (Helix 1)**								
Backbone atoms	0.5±0.2	0.5±0.2	0.4±0.2	0.3±0.1	0.07±0.10	0.01±0.01	0.16±0.06	0.14±0.07
All heavy atoms	1.7±0.4	1.5±0.3	1.2±0.2	1.0±0.2	0.9±0.4	0.4±0.1	0.8±0.2	0.8±0.2
**Residues 16-27 (Helix 2)**								
Backbone atoms	3.4±0.9	3.5±1.5	3.3±1.3	3.4±1.3	0.03±0.02	0.06±0.04	0.17±0.09	0.08±0.04
All heavy atoms	5.3±0.9	5.3±1.4	5.1±1.3	5.0±1.1	0.7±0.2	0.2±0.1	0.5±0.1	0.6±0.2
**Ramachandran plot (%)**								
Most favoured regions	71.3	75.0	77.1	78.8	87.7	88.5	89.4	96.2
Additionally allowed regions	27.1	24.8	22.9	20.8	12.3	11.5	10.6	3.8
Generously allowed regions	1.5	0.2	0	0.4	0	0	0	0
Disallowed regions	0	0	0	0	0	0	0	0

Supplementary material

(all methods HPLC: 0-60% in 15 min)

1 - hECP30

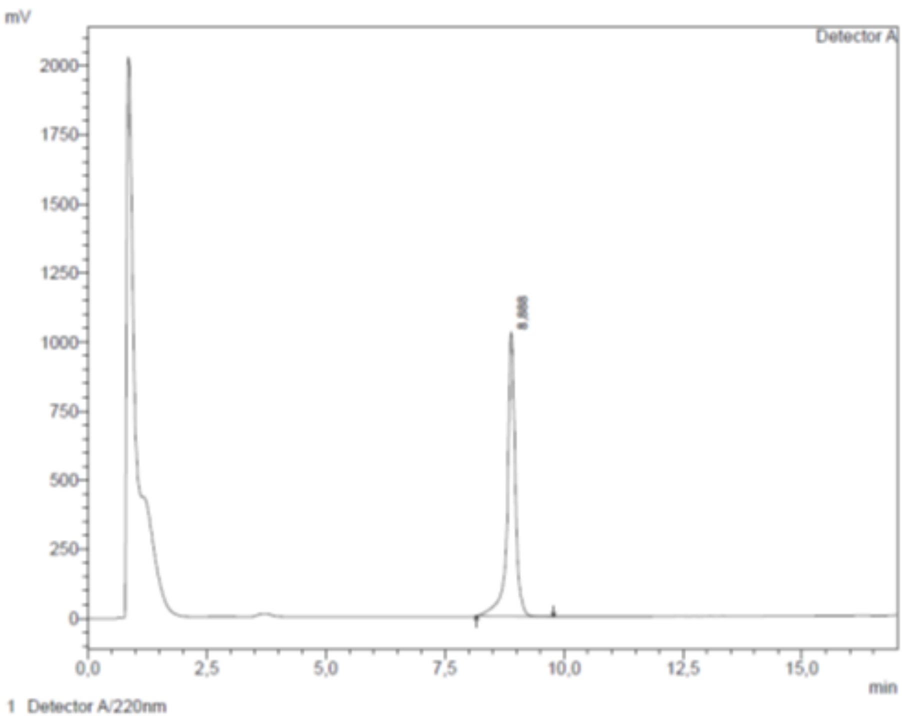

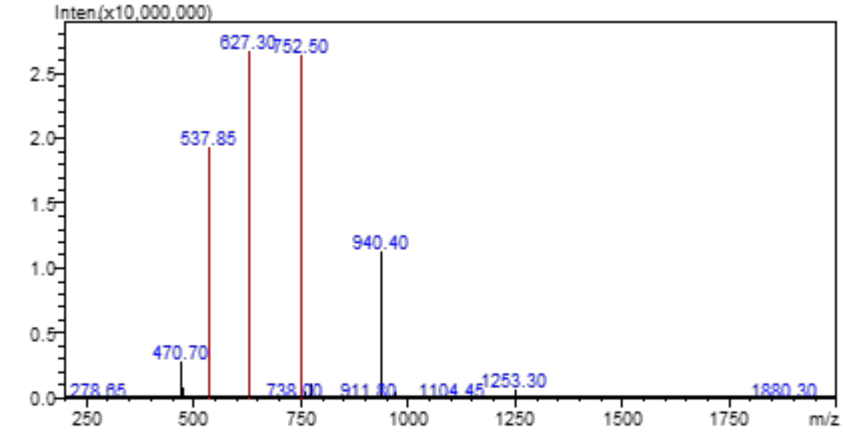

Positively charged ion series

Ch.	Average	MonoIso.
MH1+	3758.4016	3756.0193
MH2+	1879.7044	1878.5133
MH3+	1253.4720	1252.6780
MH4+	940.3559	939.7603
MH5+	752.4861	752.0097
MH6+	627.2397	626.8426
MH7+	537.7779	537.4376
MH8+	470.6816	470.3838
MH9+	418.4955	418.2308
MH10+	376.7467	376.5085

2 - Orn analog

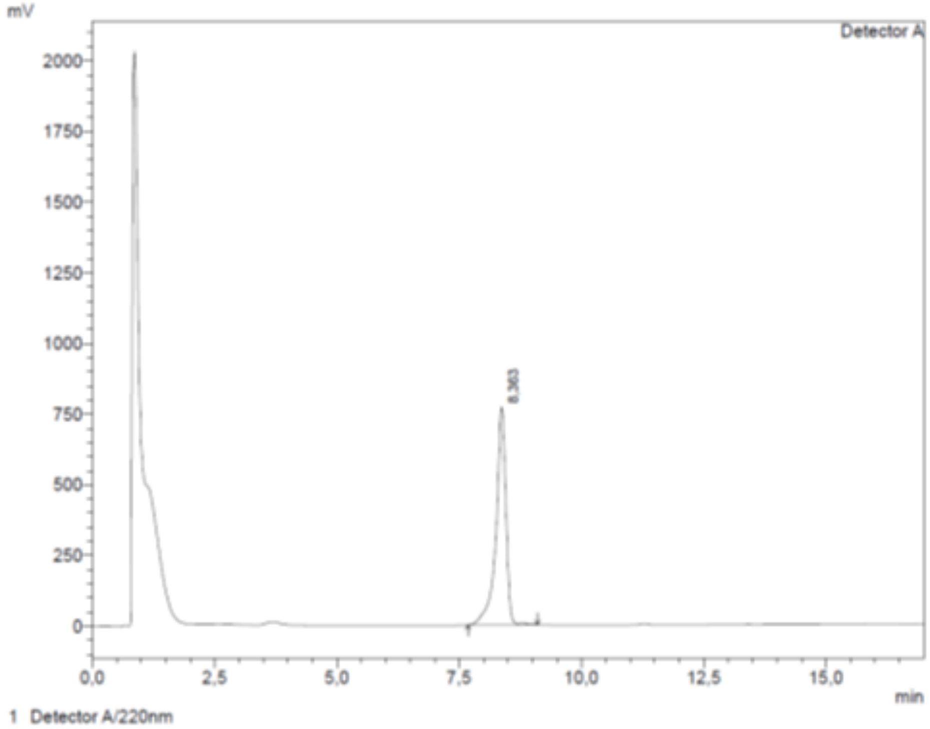

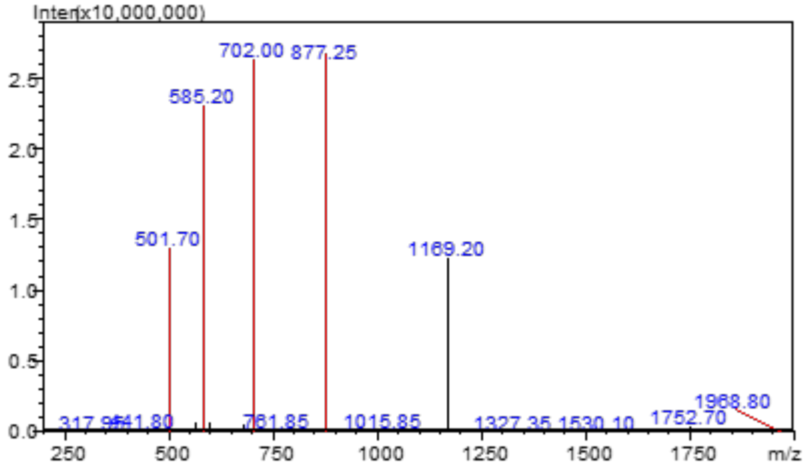

Positively charged ion series

Ch.	Average	MonoIso.
MH1+	3506.1599	3503.8885
MH2+	1753.5836	1752.4479
MH3+	1169.3915	1168.6344
MH4+	877.2954	876.7276
MH5+	702.0378	701.5835
MH6+	585.1994	584.8208
MH7+	501.7434	501.4189
MH8+	439.1514	438.8674
MH9+	390.4687	390.2163
MH10+	351.5225	351.2954

3 - Dab analog

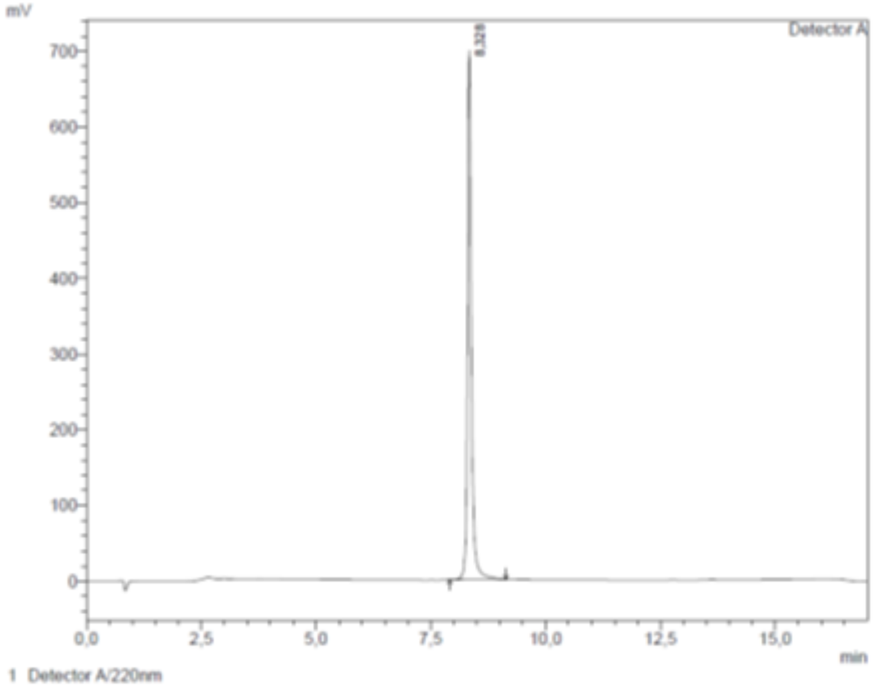
mV
700
600
500
400
300
200
100
0
Detector A
0,0
2,5
5,0
7,5
10,0
12,5
15,0
min
1 Detector A/220nm

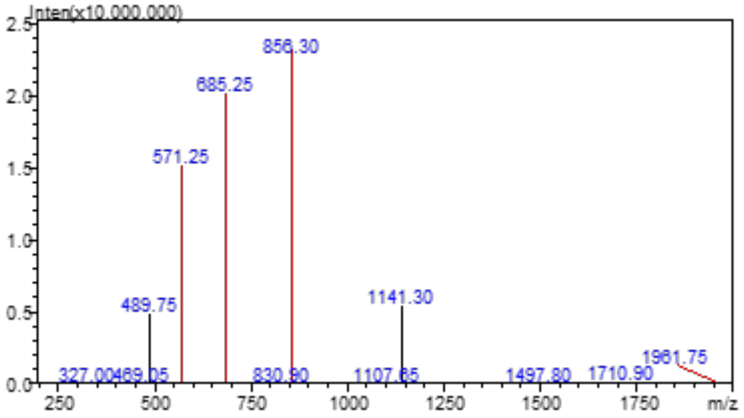
Inten(x10,000,000)
2.5
2.0
1.5
1.0
0.5
0.0
856.30
685.25
571.25
489.75
1141.30
1961.75
327.00
469.05
830.90
1107.65
1497.80
1710.90
250
500
750
1000
1250
1500
1750
m/z

Positively charged ion series

Ch.	Average	MonoIso.
MH1+	3421.9986	3419.7946
MH2+	1711.5030	1710.4009
MH3+	1141.3377	1140.6031
MH4+	856.2551	855.7041
MH5+	685.2055	684.7647
MH6+	571.1725	570.8052
MH7+	489.7203	489.4055
MH8+	428.6312	428.3557
MH9+	381.1174	380.8725
MH10+	343.1064	342.8860

4 - Har analog

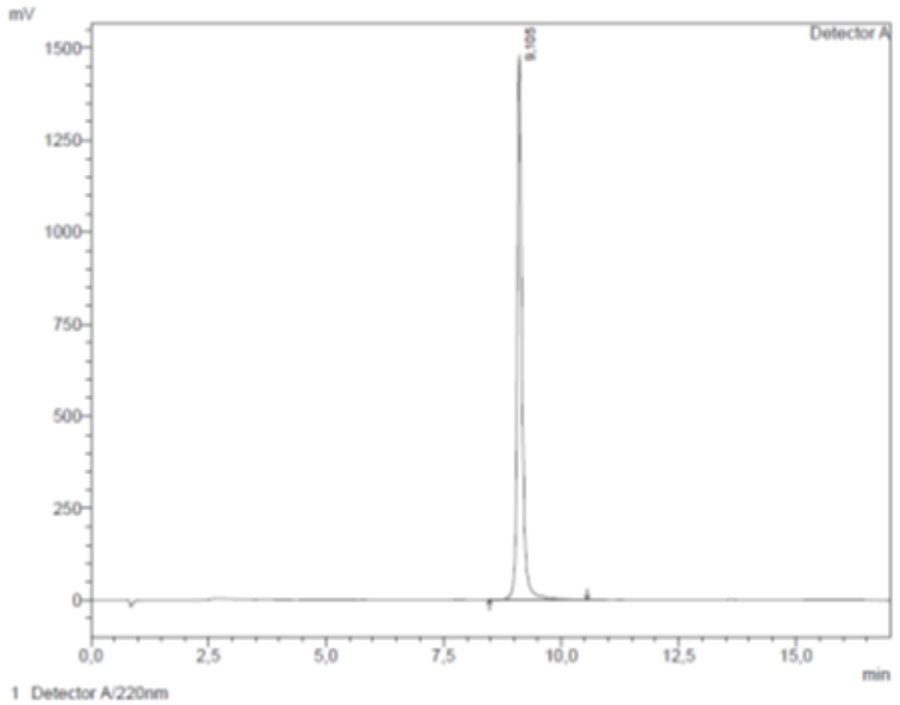

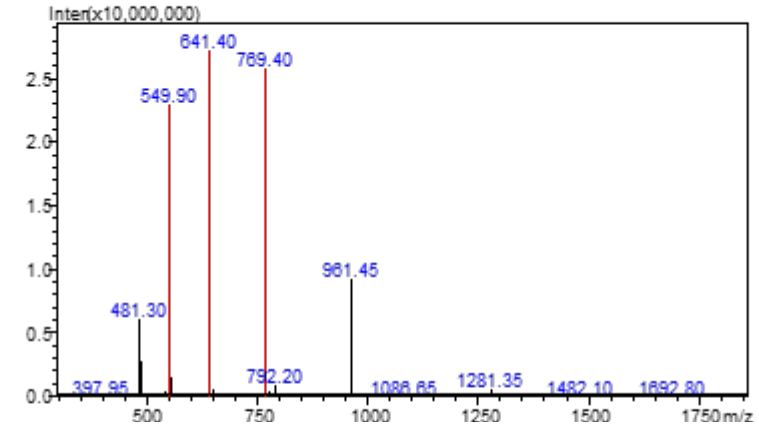

Positively charged ion series

Ch.	Average	MonoIso.
MH1+	3842.5629	3840.1132
MH2+	1921.7851	1920.5602
MH3+	1281.5258	1280.7093
MH4+	961.3962	960.7838
MH5+	769.3184	768.8285
MH6+	641.2665	640.8583
MH7+	549.8009	549.4510
MH8+	481.2017	480.8955
MH9+	427.8468	427.5746
MH10+	385.1628	384.9179

5 - Des[Orn^{1}-Pro^{2}] analog

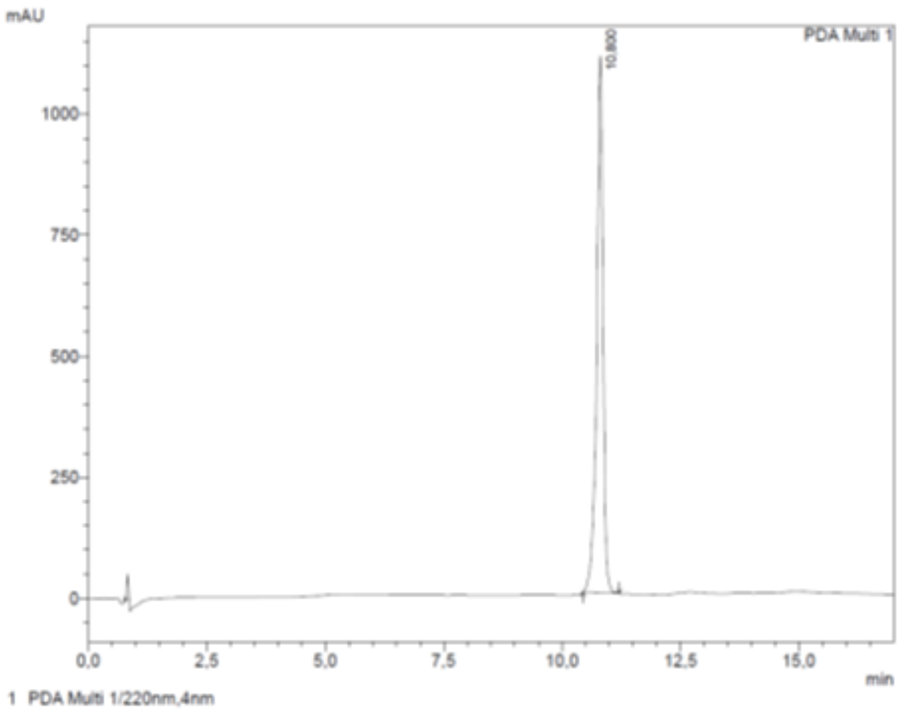

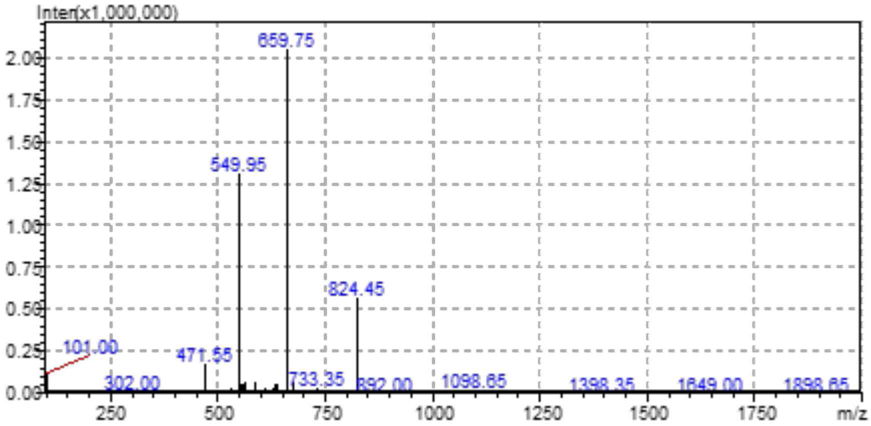

Positively charged ion series

Ch.	Average	MonoIso.
MH1+	3294.8960	3292.7564
MH2+	1647.9517	1646.8819
MH3+	1098.9702	1098.2570
MH4+	824.4795	823.9446
MH5+	659.7850	659.3571
MH6+	549.9887	549.6321
MH7+	471.5628	471.2572
MH8+	412.7434	412.4759
MH9+	366.9949	366.7572
MH10+	330.3962	330.1822

6 - [Orn[1], D-Pro[2]] analog

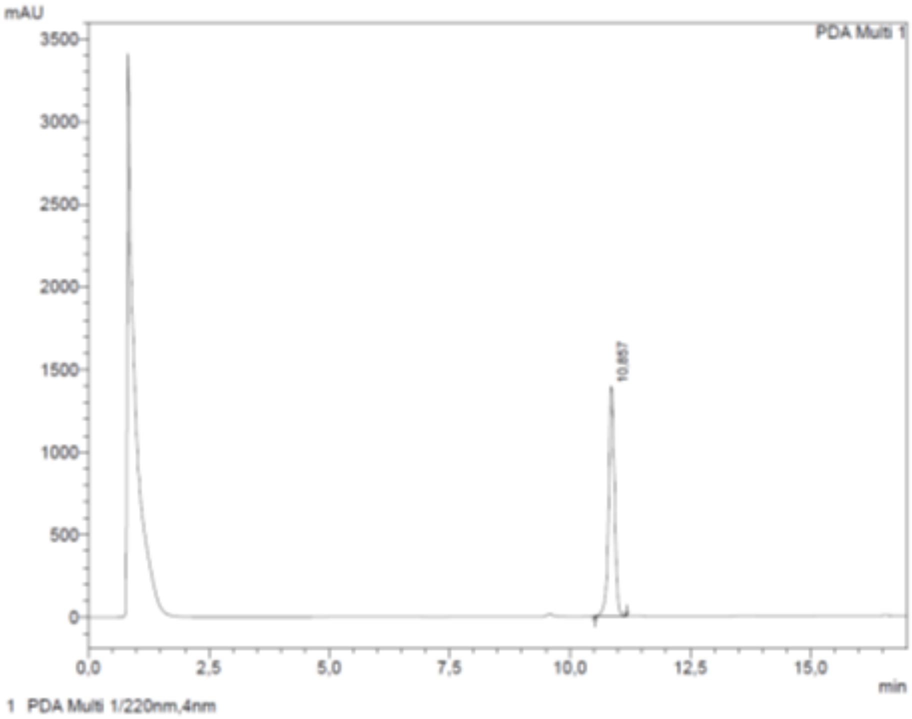

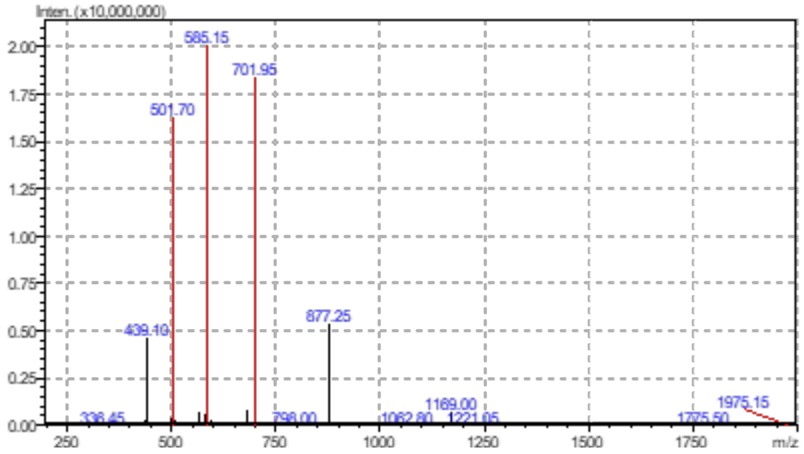

Positively charged ion series

Ch.	Average	MonoIso.
MH1+	3506.1599	3503.8885
MH2+	1753.5836	1752.4479
MH3+	1169.3915	1168.6344
MH4+	877.2954	876.7276
MH5+	702.0378	701.5835
MH6+	585.1994	584.8208
MH7+	501.7434	501.4189
MH8+	439.1514	438.8674
MH9+	390.4687	390.2163
MH10+	351.5225	351.2954

7 - [D-Orn[1], D-Pro[2]] analog

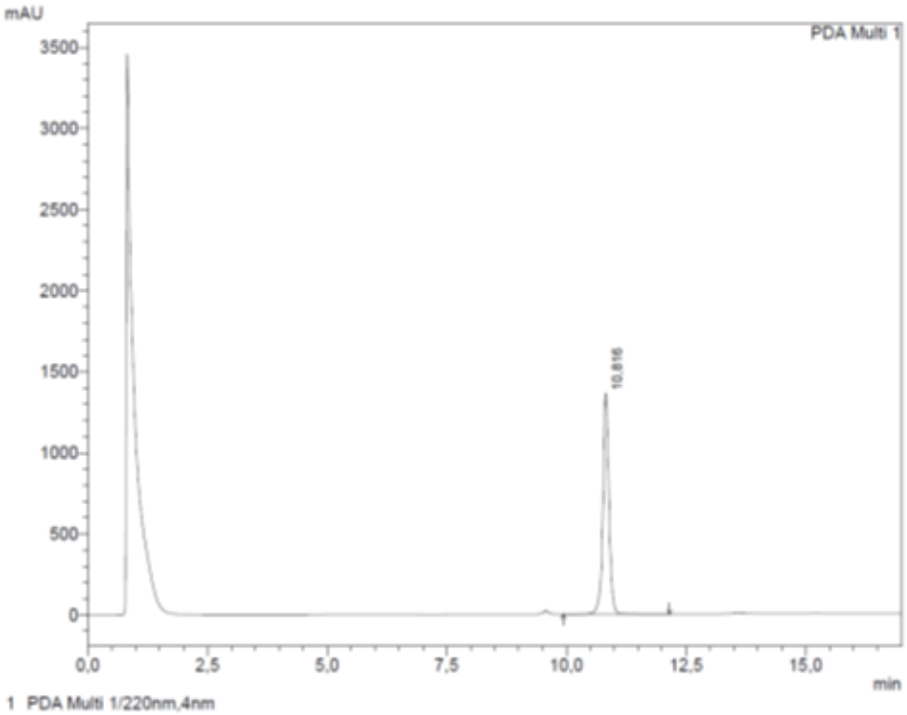

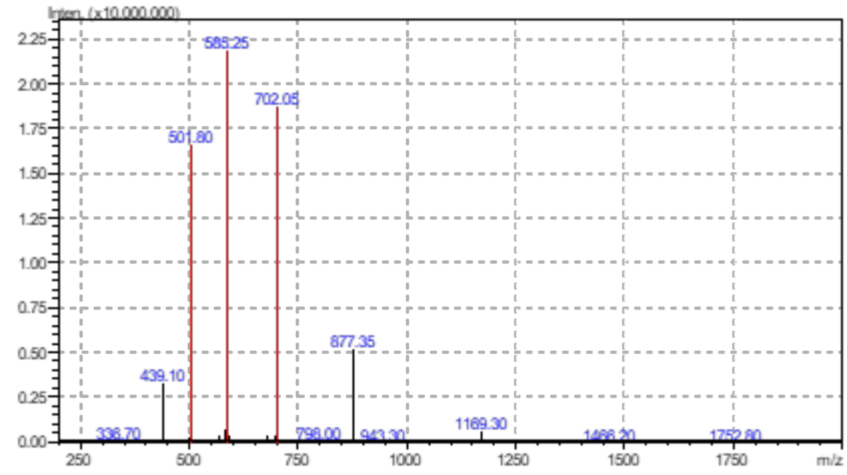

Positively charged ion series

Ch.	Average	MonoIso.
MH1+	3506.1599	3503.8885
MH2+	1753.5836	1752.4479
MH3+	1169.3915	1168.6344
MH4+	877.2954	876.7276
MH5+	702.0378	701.5835
MH6+	585.1994	584.8208
MH7+	501.7434	501.4189
MH8+	439.1514	438.8674
MH9+	390.4687	390.2163
MH10+	351.5225	351.2954
